# Elevated atmospheric CO_2_ has small, species-specific effects on pollen chemistry and plant growth across flowering plant species

**DOI:** 10.1038/s41598-024-63967-z

**Published:** 2024-06-14

**Authors:** Olivia M. Bernauer, Anupreksha Jain, Benjamin de Bivort, N. Michele Holbrook, Samuel S. Myers, Lewis H. Ziska, James D. Crall

**Affiliations:** 1 Department of Entomology, University of Wisconsin-Madison, 1630 Linden Drive, Madison, WI 53706 USA; 2 Department of Organismic and Evolutionary Biology, Harvard University, 52 Oxford Street, Cambridge, MA 02138 USA; 3 Bloomberg School of Public Health, Johns Hopkins University, 615 N Wolfe St, Baltimore, MD 21205 USA; 4 Mailman School of Public Health, Columbia University, 722 W. 168Th Street, New York, NY 10032 USA

**Keywords:** Global change, Pollen protein, Pollen nutrition, Pollinator nutrition, Climate change, Plant-pollinator interactions, Climate-change impacts, Phenology, Ecosystem services, Natural variation in plants, Plant ecology, Plant physiology, Secondary metabolism

## Abstract

Elevated atmospheric carbon dioxide (eCO_2_) can affect plant growth and physiology, which can, in turn, impact herbivorous insects, including by altering pollen or plant tissue nutrition. Previous research suggests that eCO_2_ can reduce pollen nutrition in some species, but it is unknown whether this effect is consistent across flowering plant species. We experimentally quantified the effects of eCO_2_ across multiple flowering plant species on plant growth in 9 species and pollen chemistry (%N an estimate for protein content and nutrition in 12 species; secondary chemistry in 5 species) in greenhouses. For pollen nutrition, only buckwheat significantly responded to eCO_2_, with %N increasing in eCO_2_; CO_2_ treatment did not affect pollen amino acid composition but altered secondary metabolites in buckwheat and sunflower. Plant growth under eCO_2_ exhibited two trends across species: plant height was taller in 44% of species and flower number was affected for 63% of species (3 species with fewer and 2 species with more flowers). The remaining growth metrics (leaf number, above-ground biomass, flower size, and flowering initiation) showed divergent, species-specific responses, if any. Our results indicate that future eCO_2_ is unlikely to uniformly change pollen chemistry or plant growth across flowering species but may have the potential to alter ecological interactions, or have particularly important effects on specialized pollinators.

## Introduction

Carbon dioxide (CO_2_), which continues to rise, is recognized as a major greenhouse gas contributing to climate change^1^. CO_2_ is also the sole supplier of carbon (C) for plant photosynthesis, and as such, its ongoing increase directly affects plant productivity, essentially providing ‘C-fertilization’. Rising CO_2_ levels, for example, can alter plant growth and development^2–4^, chemistry^5,6^, and reproduction and floral biology^7,8^. Increasing CO_2_ and corresponding carbon availability to plants leads to an increase in carbon-based compounds and a decrease in the concentration of compounds rich in other elements, such as nitrogen-rich proteins^9^. While a decrease in protein has been observed across multiple plant species and tissue types^10–12^, some plants may maintain sufficient nitrogen (N) levels and avoid becoming N-limited (i.e., N-fixing legumes), ultimately leading to no or little reductions in protein content^13^. This suggests that life history traits may influence the degree to which plant species are impacted by elevated CO_2_ (eCO_2_) levels.

Initial findings suggest that eCO_2_ can also affect the plant nutritional rewards for pollinators, which can, in some cases, rely exclusively on floral resources^8^. For nectar, eCO_2_ can increase or decrease nectar quantity in some plant species, though for most studied plants, nectar sugar content was not affected by increasing CO_2_^14–16^. Notably, changes to pollen nutrition in response to eCO_2_ are largely unknown. Ziska et al.^17^ demonstrated that elevated atmospheric CO_2_ levels (280–500 ppm) reduced pollen protein content in goldenrod (Asteraceae: *Solidago canadensis* L.) in both historical specimens and experiments simulating historic and near-future CO_2_ scenarios. Surprisingly, even short-term exposure (24 h) to eCO_2_ (500, 1000, and 3000 ppm) in black maple (Sapindaceae: *Acer negundo* L.) resulted in reduced soluble pollen protein levels^18^. However, in the same study on black maple, the authors found the opposite trend of increased pollen protein with 6 h of exposure to eCO_2_^18^, demonstrating the complexity of plant responses to eCO_2_. Because bees and many other pollinating insects rely on pollen as their primary source of protein, reduced pollen protein under eCO_2_ could significantly affect pollinator nutrition and health^19–21^, with subsequent impacts on pollinator populations and pollination services^22^. However, we have little understanding of the generality of these changes in floral nutrition as few flowering species have been studied under eCO_2_.

Just as changes to atmospheric CO_2_ can alter plant protein levels, eCO_2_ is likely to affect the secondary metabolites and amino acids found in pollen (and in other plant tissues^23^), with potential knock-on effects for pollinators. Some secondary metabolites can, for example, facilitate learning and memory formation in bees (e.g., caffeine^24^), and the amino acid content of pollen can structure bee foraging preferences^25,26^. Pollen is chemically complex, and possible effects of most of their constituent compounds on flower visitors are unknown. Still, research on ragweed (Asteraceae: *Ambrosia artemisifolia* L.) pollen found that eCO_2_ can alter the expression of secondary plant metabolites^27^ and has been shown to increase flavonoid metabolites^28^. Flavonoids are largely responsible for color production in plant tissues, which can facilitate plant-pollinator interactions by attracting pollinators or signaling when flowers have already been pollinated^29^. As the importance of secondary metabolites and amino acids are elucidated for pollinators, understanding if and how these compounds change in composition and abundance under a future climate with elevated CO_2_ levels is required.

For vegetative tissues, eCO_2_ growing conditions typically result in increased growth^4^. Leaves increase in both biomass and area under eCO_2_ growing conditions^30,31^ and plants grow taller, increasing above-ground biomass with exposure to eCO_2_^30^. Similarly, plants generally produce more flowers, fruits, and seeds when grown under increasing CO_2_ levels^7^. However, Jablonski et al.^7^ found that response to eCO_2_ varied between crop and wild plant species, with crop plants exhibiting a greater increase in fruits and seeds than wild species when exposed to eCO_2_. A recent review on eCO_2_ (mean eCO_2_ of 730 ppm vs mean ambient CO_2_ (aCO_2_) of 360 ppm) and flowering biology found that plants flower nearly four days earlier on average under eCO_2_^8^. For organisms that rely on plant floral resources, like pollinators, changes to plant growth patterns or flowering phenology could disrupt plant-pollinator interactions, potentially leading to reduced phenological overlap between plants and pollinators^32^.

Here, we conducted two greenhouse experiments to evaluate how eCO_2_ growing conditions impact pollen chemistry (nutrition as measured by pollen %N a direct proxy for protein content^33^, secondary metabolites, and amino acids) and plant growth in fourteen flowering plant species spanning nine plant families and two pairs of functional groups (1): plants that can and cannot fix nitrogen and 2: crop and non-crop plants). We predicted that eCO_2_ growing conditions: 1) will reduce pollen nutrition (%N) in plant species that cannot fix N due to limited N availability, but would observe no changes in pollen %N in N-fixing plant species which have greater access to N through symbiotic microbes; (2) will alter the content of amino acids and secondary metabolites in pollen^34^; (3) plants would grow taller with more leaves as increased CO_2_ increases plant growth rates^2^, but that flower number will not differ between treatments^8,16^; and 4) that plants will flower earlier when compared to plants grown in aCO_2_^8^.

## Methods

Two greenhouse studies were conducted to characterize the impacts of eCO_2_ on pollen chemistry and plant growth in several species of flowering plants. The first study was conducted in Cambridge, MA (USA), and quantified the impacts of eCO_2_ on pollen or anther chemistry (nutrition, secondary metabolites, and amino acids); if flowers were small, whole anthers were collected and analyzed as a proxy for pollen in some species (as in Ziska et al.^17^). The second study was conducted in Madison, WI (USA), and quantified impacts on pollen or anther chemistry (nutrition) and plant growth. Focal plant species across these experiments were selected for (a) their importance as forage plants for bees across a range of crop and non-crop flowering plants, (b) taxonomic diversity, and (c) functional diversity (species with root-symbiotic N-fixing bacteria vs non-N-fixing species; crop vs non-crop plant species) (Table 1). Both greenhouse studies were conducted in compliance with relevant institutional, national, and international guidelines and legislation.

**Table 1:** Description of plants used in each study and which data was collected for each plant species.

Family	Scientific name	Common name	Functional traits		Experiment(s)	Seed source	Number of plants planted per experiment)	Variables measured				Pollinators supported
			N-fixer	Crop species				Pollen nutrition (%N, %C, C:N)	Pollen secondary chemistry and amino acids	Growth (height, leaves, biomass, flowers)	Flowering characteristics (diameter, initiation)	
Asteraceae	*Helianthus annuus* L. var Domino	sunflower		*	1	AM	60	1	1			Bees, flies, butterflies, beetles^36^
	*Helianthus annuus* L. var. Yellow Pygmy	sunflower		*	2	AM	96	2		2	2	
	*Taraxacum officinale* F. H	dandelion			2	SN	48			2		Bees, flies, butterflies^37,38^
Boraginaceae	*Borago officinalis* L	borage			2	AM	96	2		2	2	Bees, flies^39,40^
	*Phacelia tanacetifolia* Benth	lacy phacelia			2	AM	96	2		2	2	Bees, flies, beetles, butterflies, wasps^41–43^
Brassicaceae	*Lobularia martimia* (L.) Desy	sweet alyssum			2	AM	96	2		2	2	Bees, flies, beetles, butterflies, wasps ^43–45^
Cucurbitaceae	*Cucumis melo* L. var Green Nutmeg	melon		*	1	SC	60	1				Bees^46,47^
	*Cucurbita pepo* L. var yellow crookneck	squash		*	1	SSE	60	1	1			Bees^48^
Fabaceae	*Trifolium pratense* L	red clover	*		2	AM	96	2		2	2	Bees^49–51^
	*Chamaecrista fasiculata* (Michx.) Greene	partridge pea	*		2	AM	48			2	2	Bees, wasps^52^
Papaveraceae	*Eschscholzia californica* Cham	poppy			1	AM	60	2	1			Bees, flies, beetles, butterflie^53–55^
Polygonaceae	*Fagopyrum esculentum* Moench	buckwheat			1, 2	AM	60, 96	2	1	2	2	Bees, flies, ants, wasps^43,56–58^
Solanaceae	*Physalis ixocarpa* Brot. purple variety	tomatillo		*	1	SSE	60	1				Bees^59,60^
	*Solanum lycopersium* L. var Blondkopfchen	tomato		*	1	SSE	60	1				Bees^61^
Tropaeolaceae	*Tropaeolum majus* L	nasturtium			2	AM	96	2		2	2	Bees, flies, beetles^43,62,63^

For functional traits, an asterisk (*) in either column indicates a species that exhibits that functional trait (i.e., can fix N, is a crop species). For each of the variables measured columns, if a variable was measured for a specific species, this is denoted with a 1 or 2 corresponding to the experiment this variable was measured in. The “Seed Source” column lists the company where seeds were sourced from: American meadows (AM), seed needs (SN), seeds of change (SC), and seed savers exchange (SSE).

### Experiment 1-experimental design

We grew plants from seed (source detailed in Table 1) in two adjacent greenhouse chambers each 42.2 m^2^ in size at Harvard University (Cambridge, MA, USA), one aCO_2_ and one eCO_2_ (elevated to a target of 650 ppm CO_2_) chamber. 650 ppm CO_2_ was chosen as our eCO_2_ treatment as this was approximately 200 ppm above current aCO_2_ levels and within the predicted CO_2_ range for 2100^35^; CO_2_ treatments were initiated from planting. Two experimental rounds were conducted with CO_2_ treatments switching chambers between rounds to account for chamber effects (round 1 was planted in September 2020 and round 2 in January 2021). We planted between 15 plants of each focal species per chamber per round (n = 60 total plants per species; Table 1).

Focal species included (1) buckwheat, (2) melon, (3) poppy, (4) sunflower, (5) tomatillo, (6) tomato, and (7) squash (Table 1). Plants were grown from seed in 6-inch (15.2 cm) diameter pots under 16:8 light:dark photoperiod with 23 and 17 °C day and night temperatures, respectively. Ventilation was provided by manually operated vents and plants were grown in Premier Pro-Mix BX 3.8 ft^3^/bale with mycorrhizae and provided slow-release fertilizer (1.8 kg/m^3^ 14-14-14 Osmocote with a 3–4 month longevity). Plants were watered until water dripped through the pots either every day or every other day and treated with systemic pesticides for thrips as needed throughout the study.

One greenhouse chamber served as the ambient CO_2_ (aCO_2_) chamber. In the second chamber, CO_2_ was elevated to a target of 650 ppm (eCO_2_). CO_2_ levels were maintained in the eCO_2_ room using a LiCor Li-850 gas analyzer and Arduino-based controller. The LiCor Li-850 monitored CO_2_ levels, relaying this to the Arduino-based controller which, if low (50 ppm or more below the target CO_2_ levels), would initiate flow from a CO_2_ tank (AirGas; via a solenoid-operated flow regulator) to maintain the target CO_2_ concentration. If CO_2_ levels were at or above 650 ppm, the Arduino-based controller would not initiate CO_2_ flow. Observed median CO_2_ concentrations in the elevated CO_2_ chambers were 647 and 642 ppm for experimental rounds 1 and 2, respectively (Fig. S1a). CO_2_ concentrations were quantified in the aCO_2_ chambers using an identical LiCor Li-850 gas analyzer. Median CO_2_ concentrations in the uncontrolled chamber were 445 and 438 ppm for rounds 1 and 2, respectively (Fig. S1a).

### Experiment 1–pollen collection and processing

In both rounds, pollen and/or anthers (a proxy for pollen^17^; hereafter referred to as pollen samples) were collected from flowering plants: for the two buzz-pollinated species with poricidal anthers (tomato and tomatillo), pollen was collected using a tuning fork, for melon and buckwheat, anthers were collected using forceps. For poppy, sunflower, and squash, the flowers were gently shaken over weighing paper as they readily dropped pollen. We began collecting pollen samples when plants started flowering and continued to collect pollen throughout bloom, up to five times per individual plant; therefore, pollen samples contain a mix of pollen throughout bloom stages. Pollen samples were collected into a new microcentrifuge tube during each collection event and stored in a −80 °C freezer after collection. Once pollen was collected, samples were pooled across days by individual plant. Pollen samples were allocated for two analyses: targeted metabolomics, where pollen samples were kept frozen and analyzed fresh, and elemental C and N analyses, where pollen samples were dried in a drying oven at 50 °C for 48 h.

### Experiment 2–experimental design

We grew plants from seed (source detailed in Table 1) in four 10.2 m^2^ greenhouse chambers on the campus of the University of Wisconsin–Madison with two chambers experiencing aCO_2_ and two with eCO_2_ (with a target of 690 ppm, selected as a realistic 2100 prediction^35^). We systematically assigned CO_2_ treatments to the four greenhouse chambers alternating between eCO_2_ and aCO_2_. Two experimental rounds were conducted to increase sample size and account for chamber effects, though because rounds 1 and 2 overlapped, chambers were not switched between rounds (round 1 was planted in November 2021 and round 2 in January 2022). We planted 12 pots per focal plant species per greenhouse chamber per round (total plants per species detailed in Table 1) and eCO_2_ was initiated 14 days after seeds were planted.

Nine flowering plant species with known value to pollinating insects were studied (Table 1): (1) buckwheat, (2) borage, (3) lacy phacelia, (4) red clover, (5) partridge pea, (6) nasturtium, (7) dwarf sunflower, (8) dandelion, and (9) sweet alyssum. All pots (2 in^3^, or 0.3 L) were sown with at least two seeds and thinned to the strongest seedling on day 14. Plants were re-potted to 6 in (15.2 cm) diameter, 1.9 L, pots on an as-needed basis (i.e., roots growing out of the bottom of the pot) and watered until water dripped through the pots either every day or every other day. Pests were monitored by greenhouse staff; plants were treated for thrips two times throughout the study (once with Hachi-Hachi, Radiant, and Pradia and once with Pylon and Talstar).

In each greenhouse, lighting was set to a 16:8 light:dark photoperiod, and temperatures were set to a minimum of 18 °C during the day, and 15 °C at night. If chambers were experiencing temperatures greater than 27 °C, cooling was automatically provided by vents in the side and ceiling of each chamber. All seeds were sown into ProMix HP Biofungicide + Mycorrhizae medium with a medium feeding rate (84 g/sq ft. of soil) of controlled-release fertilizer (Nutricote Total, 13-11-11, Type 100). Two greenhouse chambers were unmanipulated and served as the aCO_2_ chambers while in the remaining two chambers, CO_2_ was elevated to a target of 690 ppm for the eCO_2_ treatment using the same setup as in experiment 1. Additionally, in each chamber, a CO_2_ sensor (Adafruit SCD-30, run by Arduino UNO) collected a reading every ten minutes. For the eCO_2_ chambers, observed median CO_2_ concentrations were 645 ppm in both rounds and the aCO_2_ chambers had observed median CO_2_ levels of 456 and 443 for rounds 1 and 2, respectively (Fig. S1b).

### Experiment 2–plant growth data collection

Starting on day 14, and every 7 days thereafter, the number of leaves and the plant’s height was collected for each individual plant. Once plants began producing flowers, flower numbers were recorded daily. Due to time constraints, we stopped counting flowers for sweet alyssum after week 10 (when a plant had > 500 flowers) and buckwheat after week 11 (when a plant had > 100 flowers).

Flower diameter data was collected from five open flowers (or inflorescences in the case of red clover and sunflower) per individual plant for all species except sunflower which only produced a single inflorescence per plant. We measured flower diameter within the first three days of a flower being open. For each flower, three diameter measurements were taken and then these were averaged per flower, then the flower diameter values were averaged for each individual plant, resulting in one flower diameter value per plant. We collected flower diameter data for a minimum of five plants per species in each greenhouse chamber and round.

As CO_2_ is known to impact plant growth, we destructively sampled above-ground biomass on day 60 for half of the plants grown in round 1 (up to 6 plants per species per greenhouse chamber). Many plants in round 1 had not yet flowered when biomass samples were collected, reducing the total possible number of pollen samples. As a major focus for this study was to investigate how pollen nutrition (in the form of %N) changed in response to elevated CO_2_, we opted to forego the destructive above-ground biomass sampling in round 2, maximizing the number of plants available for pollen sampling. To measure biomass, plants were cut at ground level, placed into a pre-weighed paper bag, and then dried at room temperature until the weight stabilized. The final measurements of dry plant biomass were obtained following another 14 days of storage at room temperature.

### Experiment 2–pollen collection and processing

One pollen or anther sample (pooled across days) was collected from freshly opened (within three days of opening) flowers per round. For borage and partridge pea flowers, we used a VegiBee Sonic Garden Pollinator to release pollen from poricidal anthers directly into microcentrifuge tubes. For buckwheat, lacy phacelia, and sunflowers, when flowers were open and producing pollen, they were held over a plastic weigh boat and gently tapped with a paintbrush, and any pollen dropped was brushed into a microcentrifuge tube. For nasturtium, red clover, and sweet alyssum, whole anthers were collected directly from flowers using fine forceps; for these plant species, we use anthers as a proxy for pollen (as in Ziska et al.^17^) and refer to these samples as pollen samples hereafter. Dandelions in this study never flowered. Pollen samples were stored in a −4 °C freezer until sample sizes were large enough for pollen to fill the bottom 3–5 mm of the microcentrifuge tube (pooled across collection days if needed), then were dried at 60 °C for 48 h in a drying oven.

### Pollen C and N analysis

To collect C and N composition data, pollen samples were flash-analyzed in the Jackson Lab at the University of Wisconsin–Madison. Pollen and anther samples were processed and analyzed in the same manner. Pollen samples were placed into tin capsules and the samples were carefully rolled to enclose all contents. Next, samples were flash combusted using a Thermo Flash EA1112 Carbon Nitrogen analyzer to obtain values for the %N and %C present in each sample. The %N value serves as an approximation for protein present within a pollen sample^33^. In experiment 1, 1.0 ± 0.5 mg samples of dried pollen were analyzed, while samples analyzed from experiment 2 contained a dry pollen mass of 1.5 ± 0.5 mg.

### Pollen secondary chemistry

Targeted metabolomics were performed on pollen or anther samples for a subset of plant species (buckwheat, sunflower, squash, tomato, and poppy) to assess how pollen secondary chemistry is affected by changing CO_2_ levels. 5 mg samples of fresh pollen were prepared following the methods in Palmer-Young et al.^64^: the pollen sample was first combined with 1 ml of methanol then incubated for 10 min in an ultrasound bath, then overnight at room temperature. Samples were centrifuged for 10 min at maximum speed and then the supernatants were transferred to a 1.5 ml glass autosampler vial. Next, samples were dried under N_2_ flow and then resuspended in methanol (at a rate of 500 ul/5 mg). Secondary chemistry was quantified using a ThermoFisher IDX LC-MS using a Kinetex F5 column at the Harvard Center for Mass Spectrometry (Cambridge, MA, USA). 5 µL of each sample was injected on a Kinetex F5 column (2.6 um, 150 × 2 mm, Phenomenex), maintained at 30 °C. Mobile phases were A: water, 2.5 mM ammonium acetate and B: Methanol:Isopropyl Alcohol 1:1. The flow rate was maintained at 0.2 mL min^-1^. The gradient was as follows: 5 min at 0% B, then to 100% B in 20 min, followed by 10 min at 100% and finally 6 min re-equilibration at 0% B. Data was acquired in MS1 for all samples, with switching polarities at 120,000 resolution and a scan range of 65 to 1000 m/z. Retention time and ions were confirmed with standards and for all targeted compounds. Tracefinder (ThermoFisher Scientific) was used to integrate the corresponding peaks in each sample.

### Data analysis

Statistical analyses were carried out in R v. 4.2.2^65^. For both experiments, we included plant species in our analyses that had at least three data points per treatment.

When we used models (linear models, linear mixed effect models, generalized linear mixed models) to compare the effects of CO_2_ on pollen chemistry or plant growth, model fit was evaluated using the package *DHARMa*^66^. For linear models, the statistical significance of the fixed effects was assessed using a Type III Analysis of Variance with Satterthwaite’s method using the function “anova” with the package *lmerTest*^67^ to evaluate main and interactive effects for model terms. We report significant fixed effects using Type II analysis of deviance (Type II Wald Chi-Square tests) for generalized linear mixed-effect models. Specific models for each variable measured are outlined below. To investigate species-specific responses to CO_2_ treatment, we used post-hoc pairwise comparisons (function “pairs” in *emmeans*^68^).

In the text of our results, alongside significant raw p-values, we report the strength of effects using the language of evidence (per Fig. 1 of Muff et al.^69^): little or no evidence (1 > p > 0.1), weak evidence (0.1 > p > 0.05), moderate evidence (0.05 > p > 0.01), strong evidence (0.01 > p > 0.001), and very strong evidence (0.001 > p). Where little or no evidence or weak evidence (p > 0.05) was found, we do not report the p-value in text and only report the relevant language of evidence^69^.

**Figure 1:**
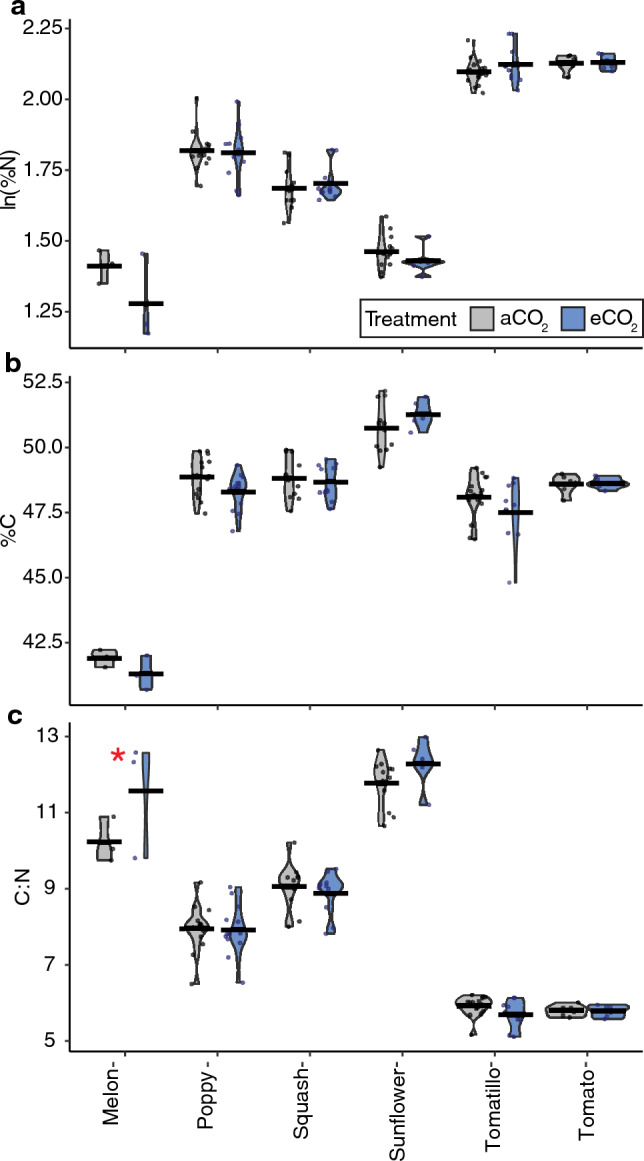
Impacts of elevated CO_2_ on pollen nutrition in experiment 1. Pollen nutrition from samples collected during experiment 1 including %N (**a**), %C (**b**), and C:N ratio (**c**) across CO_2_ treatments (aCO_2_: gray, eCO_2_: blue). Data from rounds 1 and 2, when available, have been pooled in this figure. Significant differences across CO_2_ treatments are denoted with a red asterisk (*). Model summaries are in Table 2, effect sizes in Table S1, and species-specific post-hoc tests in Table S2.

*Experiment 1.* After omitting small sample sizes (plant species with fewer than three data points per treatment), we analyzed 110 pollen samples from six plant species grown in experiment 1. For melon (pollen samples: eCO_2_: n = 3, aCO_2_: n = 3), poppy (pollen samples: eCO_2_: n = 13, aCO_2_: n = 14), sunflower (pollen samples: eCO_2_: n = 6, aCO_2_: n = 12), tomatillo (pollen samples: eCO_2_: n = 9, aCO_2_: n = 16), and tomato plants (pollen samples: eCO_2_: n = 6, aCO_2_: n = 6), we only had sufficient pollen samples from round 1 to analyze, while for squash, we had enough samples from rounds 1 and 2 to analyze both (pollen samples: eCO_2_: n = 11, aCO_2_: n = 11). To evaluate the effects of CO_2_ on pollen nutrition (%N, %C, and C:N), we used linear models (“lm” in the package *lme4*^70^) with CO_2_ treatment, round, plant species, and greenhouse chamber as fixed effects and evaluated the interaction between CO_2_ treatment and plant species (i.e., lm(%N ~ CO_2_* Plant_species + Round + Chamber)).

In total, 119 pollen samples were analyzed for changes to secondary compounds using targeted metabolomics, including 67 samples from round 1 (buckwheat: aCO_2_: n = 5, eCO_2_: n = 5; poppy: aCO_2_: n = 10, eCO_2_: n = 10; squash: aCO_2_: n = 4, eCO_2_: n = 3; sunflower: aCO_2_: n = 10, eCO_2_: n = 8; tomato: aCO_2_: n = 6, eCO_2_: n = 6) and 52 samples from round 2 (buckwheat: aCO_2_: n = 10, eCO_2_: n = 10; squash: aCO_2_: n = 8, eCO_2_: n = 9; sunflower: aCO_2_: n = 8, eCO_2_: n = 7). To evaluate changes to pollen secondary chemistry using targeted metabolomics, we performed principal components analysis on nine amino acids (arginine, histidine, isoleucine + lycine, lysine, methionine, phenylalanine, threonine, tryptophan, and valine). Then, for each experimental round, we ran a MANOVA to determine if CO_2_ treatment, plant species, or the interaction between the two influenced amino acid composition. In round 1, samples from tomato and squash were lacking data for several amino acids and could not be included in amino acid analyses. For the remaining secondary metabolites, we analyzed changes in each metabolite separately since many metabolites were only detected in a subset of plant species. Details on which plant species and experimental round(s) were analyzed for each compound are provided alongside the model outputs. To investigate changes in metabolites across CO_2_ treatments and plant species, we used a t-test (for nicotine, only detected in buckwheat pollen in round 1) or linear models (for caffeine, chlorogenic acid, cinnamic acid, eugenol, gallic acid, kaempferol, p-coumaric acid, and quercetin). The linear models used to evaluate secondary metabolites included CO_2_ treatment, plant species, experimental round (where possible), and greenhouse chamber as fixed effects and investigated the interaction between CO_2_ treatment and plant species (i.e., lm(gallic_acid ~ CO_2_*Plant_species + Round + Chamber)). To improve data normality, the values of caffeine, chlorogenic acid, cinnamic acid, kaempferol, p-coumaric acid, and quercetin were natural-log-transformed before analysis.

*Experiment 2.* We collected two rounds of plant growth data for borage, buckwheat, red clover, lacy phacelia, nasturtium, sweet alyssum, and sunflower. Partridge pea exhibited high mortality rates during round 2, thus only data from round 1 were analyzed. Dandelion did not flower during our experiment and, as a result, were also removed from round 2. However, data on dandelion growth (height, biomass, leaf number) are included from round 1.

We analyzed 328 pollen samples across eight species in experiment 2 after omitting groups with small samples: borage (eCO_2_: n = 23, aCO_2_: n = 25), buckwheat (eCO_2_: n = 27, aCO_2_: n = 25), red clover (eCO_2_: n = 14, aCO_2_: n = 12), lacy phacelia (eCO_2_: n = 29, aCO_2_: n = 32), nasturtium (eCO_2_: n = 20, aCO_2_: n = 19), sweet alyssum (eCO_2_: n = 15, aCO_2_: n = 23), and sunflower (eCO_2_: n = 33, aCO_2_: n = 31).

For biomass, we analyzed 194 samples for borage (eCO_2_: n = 11, aCO_2_: n = 10), buckwheat (eCO_2_: n = 10, aCO_2_: n = 11), red clover (eCO_2_: n = 11, aCO_2_: n = 12), dandelion (eCO_2_: n = 11, aCO_2_: n = 12), lacy phacelia (eCO_2_: n = 12, aCO_2_: n = 12), nasturtium (eCO_2_: n = 10, aCO_2_: n = 12), partridge pea (eCO_2_: n = 7, aCO_2_: n = 6), sweet alyssum (eCO_2_: n = 12, aCO_2_: n = 12), and sunflower (eCO_2_: n = 12, aCO_2_: n = 11).

We analyzed 380 samples for flowering start date, in analyses borage (eCO_2_: n = 21, aCO_2_: n = 22), buckwheat (eCO_2_: n = 36, aCO_2_: n = 34), red clover (eCO_2_: n = 23, aCO_2_: n = 15), lacy phacelia (eCO_2_: n = 24, aCO_2_: n = 22), nasturtium (eCO_2_: n = 24, aCO_2_: n = 17), partridge pea (eCO_2_: n = 5, aCO_2_: n = 6), sweet alyssum (eCO_2_: n = 45, aCO_2_: n = 29), and sunflower (eCO_2_: n = 29, aCO_2_: n = 28).

Flower diameter analyses included measurements from 349 plants borage (eCO_2_: n = 21, aCO_2_: n = 22), buckwheat (eCO_2_: n = 27, aCO_2_: n = 28), red clover (eCO_2_: n = 24, aCO_2_: n = 17), lacy phacelia (eCO_2_: n = 22, aCO_2_: n = 20), nasturtium (eCO_2_: n = 18, aCO_2_: n = 16), partridge pea (eCO_2_: n = 5, aCO_2_: n = 3), sweet alyssum (eCO_2_: n = 33, aCO_2_: n = 34), and sunflower (eCO_2_: n = 31, aCO_2_: n = 28).

For analyses on pollen nutrition (%N, %C, and C:N), biomass, flowering start date, and flower diameter, for which we have one data point per individual plant, we used linear models (“lm” in the package *lme4*^70^) with CO_2_, plant species, round, and greenhouse chamber as fixed effects and evaluated the interaction between CO_2_ treatment and plant species (i.e., lm(%N ~ CO_2_*Plant_species + Round + Chamber)). Flowering start date and flower diameter were natural-log transformed prior to performing linear model analyses.

Flower number, height, and leaf number data were collected multiple times on the same individual plant (each week for height and leaf number, daily from flowering onset for flower number) and for these data sets, to account for plant growth over time, we additionally include a temporal measure in analyses: weeks since planting. To account for variation within individual plants, we also include individual plant identity (a unique identifier for each plant) as a random effect in our models.

To evaluate trends in flower number under eCO_2_, we used a subset of our data, only including one data point per individual plant per week (the value on day 7 and every seven days after that). Since flower number was zero-inflated count data, we used a generalized linear mixed model with a negative binomial distribution (“glmer.nb” in *lme4*^70^) to evaluate CO_2_, plant species, round, and greenhouse chamber as fixed effects and weeks since planting as a random effect, we also investigated whether there was an interaction between CO_2_ treatment and plant species (glmer.nb(flowers ~ CO_2_*Plant_species + Round + Chamber + (1|Week)); when we included individual plant identity in our model to evaluate flower number, the model did not converge, therefore, we removed this random effect from the model.

For plant height and leaf number, data were natural log-transformed (i.e., ln(height)) to normalize the data sets. Then, evaluated using linear mixed-effect models with CO_2_, plant species, round, weeks since planting, and greenhouse chamber as fixed effects along with the interaction between CO_2_ treatment and plant species and individual plant identity as a random effect (i.e., lmer(ln(height) ~ CO_2_*Plant_species + Round + Week + Chamber + (1|Plant_identity))).

*Functional group comparisons.* We were only able to analyze pollen nutrition of one species of N-fixing plant, red clover, preventing us from exploring the relationship between N-fixing plants and non-fixing plants with respect to pollen nutrition. To compare frequency of response to eCO_2_ between crop and non-crop species, we used a Fisher’s exact test.

## Results

### *Experiment 1–impacts of eCO*_*2*_* on pollen nutrition*

*Nitrogen content.* When we compared pollen %N for all plant species, we found very strong evidence that plant species predicted %N (p < 0.0001), but no evidence that experimental round, greenhouse chamber, CO_2_ treatment or the interaction between CO_2_ treatment and plant species were predictors of %N (summarized in Table 2; effect sizes in Table S1). Consistently, post-hoc tests revealed no evidence that CO_2_ affected pollen %N for any species (Fig. 1a; statistics summarized in Table S2).

**Table 2:** Pollen nutrition (%N, %C, C:N) analyses for both experiments.

Exp	Var	Model formula	Fixed effects	F-statistic	p-value	Level of evidence
1 (n = 110)	%N	lm(%N ~ CO_2_* plant_species + round + chamber)	CO_2_	F_1_ = 0.02	0.89	None
			Plant species	F_5_ = 330.29	** < 0.0001**	**Very strong**
			Round	F_1_ = 2.08	0.15	None
			Chamber	F_1_ = 0.0002	0.99	None
			CO_2_ × plant species	F_5_ = 1.11	0.36	None
	%C	lm(%C ~ CO_2_* plant_species + round + chamber)	CO_2_	F_1_ = 10.71	**0.001**	**Strong**
			Plant species	F_5_ = 154.88	** < 0.0001**	**Very strong**
			Round	F_1_ = 25.46	** < 0.0001**	**Very strong**
			Chamber	F_1_ = 0.78	0.38	None
			CO_2_ × plant species	F_5_ = 0.61	0.69	None
	C:N	lm(C:N ~ CO_2_* plant_species + round + chamber)	CO_2_	F_1_ = 0.1	0.75	None
			Plant species	F_5_ = 330.81	** < 0.0001**	**Very strong**
			Round	F_1_ = 0.1	0.75	None
			Chamber	F_1_ = 0.63	0.43	None
			CO_2_ × plant species	F_5_ = 2.66	0.03	Strong
2 (n = 328)	%N	lm(ln(N) ~ CO_2_* plant_species + round + chamber)	CO_2_	F_1_ = 0.16	0.69	None
			Plant species	F_6_ = 625.04	** < 0.0001**	**Very strong**
			Round	F_1_ = 6.69	**0.01**	**Strong**
			Chamber	F_2_ = 2.57	0.08	Weak
			CO_2_ × plant species	F_6_ = 1.69	0.12	None
	%C	lm(ln(%C) ~ CO_2_* plant_species + round + chamber)	CO_2_	F_1_ = 0.12	0.73	None
			Plant species	F_6_ = 86.3	** < 0.0001**	**Very strong**
			Round	F_1_ = 25.93	** < 0.0001**	**Very strong**
			Chamber	F_2_ = 0.97	0.38	None
			CO_2_ × plant species	F_6_ = 0.38	0.89	None
	C:N	lm(ln(C:N) ~ CO_2_* plant_species + round + chamber)	CO_2_	F_1_ = 0.12	0.72	None
			Plant species	F_6_ = 657	** < 0.0001**	**Very strong**
			Round	F_1_ = 2.73	0.1	None
			Chamber	F_2_ = 3.3	**0.04**	**Strong**
			CO_2_ × plant species	F_6_ = 1.68	0.13	None

The column Exp denotes which experiment (1 or 2) the analyses refer to and the listed n is the total number of pollen samples analyzed. The column Var denotes which pollen nutrition variable was analyzed. Effect sizes are in Table S1 and species-level comparisons are in Table S2. Bolded p-values indicate a significant finding (α = 0.05).

*Carbon content.* When all species were compared together, we found strong evidence that CO_2_ treatment (p = 0.001) and very strong evidence that plant species (p < 0.0001) and experimental round (p < 0.0001) affected %C in pollen samples, but no evidence that greenhouse chamber nor the interaction between CO_2_ treatment and plant species did (Fig. 1b; Table 2; Table S1). Post-hoc tests found no evidence for effects of eCO_2_ on pollen %C in any plant species (Table S2).

*C:N ratios.* When the C:N ratio was analyzed, we found very strong evidence that plant species (p < 0.0001) and moderate evidence that the interaction between CO_2_ and plant species (p = 0.03) affected C:N with no evidence that CO_2_ treatment, experimental round, or greenhouse chamber influenced C:N (Table 2; Table S1). For individual species, post-hoc tests found strong evidence (p = 0.01) that eCO_2_ increased C:N ratio in melon pollen (Fig. 1c; Table S2).

### Experiment 1–targeted metabolomics

**Figure 2:**
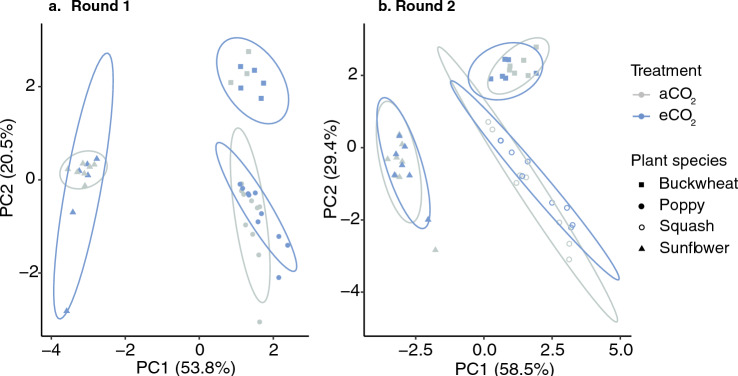
Amino acid composition in pollen across CO_2_ treatments. Principle components analysis of the pollen amino acid composition for round 1 (**a**) and 2 (**b**) for four plant species (buckwheat, poppy, squash, and sunflower) plotted against the first two principal components of amino acid variation. Note that principal components analyses were performed on each round separately so PC1 in panel a is not the same as PC1 in panel b.

In round 1, we found strong evidence that CO_2_ treatment and very strong evidence that plant species affected pollen amino acid composition, with no evidence that the interaction between CO_2_ and plant species influenced pollen amino acids (MANOVA: CO_2_ treatment: F_2, 34_ = 10.49, p = 0.0003, plant species: F_4, 70_ = 105.4, p < 0.000001; CO_2_ treatment x plant species: F_4, 70_ = 1.13, p = 0.35; Fig. 2a). When species were compared separately, we found no evidence for an effect of CO_2_ on amino acid composition (MANOVA: buckwheat: CO_2_ treatment: F_2, 5_ = 1.26, p = 0.36; poppy: CO_2_ treatment: F_2, 17_ = 3.13, p = 0.07; sunflower: CO_2_ treatment: F_2, 10_ = 1.0, p = 0.4). For round 2, we found very strong evidence for an effect of plant species on pollen amino acids, but no evidence that CO_2_ nor the interaction between the two affected pollen amino acids (MANOVA: CO_2_ treatment: F_2, 37_ = 1.72, p = 0.19, plant species: F_4, 76_ = 78.65, p < 0.000001; CO_2_ treatment x plant species: F_4, 76_ = 2.02, p = 0.1; Fig. 2b).

For specific compounds, when we consider the effect of CO_2_ on secondary metabolites, only caffeine demonstrated evidence for a weak effect of CO_2_ (p = 0.07) and two compounds, chlorogenic acid and quercetin, demonstrated strong evidence (p ≤ 0.01) for an interaction between CO_2_ treatment and plant species (Table 3; effect sizes in Table S3). Post-hoc tests (Table S4) revealed strong evidence (p = 0.001) that the amount of chlorogenic acid in buckwheat pollen was affected by CO_2_ treatment with eCO_2_ resulting in a decrease in chlorogenic acid (by 75%). For quercetin, post-hoc tests (Table S4) found strong evidence (p ≤ 0.04) that both buckwheat and sunflower pollen were affected by CO_2_ treatment, altering quercetin in pollen by −58% and + 525%, respectively.

**Table 3:** Pollen secondary metabolite analyses across plant species in experiment 1.

Variable	Plant species analyzed	Model formula	Fixed effects	Statistic	p-value	level of evidence
Caffeine (n = 108)	buckwheat^1,2^, poppy^1^, squash^1,2^, sunflower^1,2^, tomato^1^	lm(ln(caffeine) ~ CO_2_* plant_species + round + chamber)	CO_2_	F_1_ = 3.37	0.07	Weak
			Plant species	F_4_ = 5.7	**0.0004**	**Strong**
			Round	F_1_ = 61.1	** < 0.0001**	**Very strong**
			Chamber	F_1_ = 8.57	**0.004**	**Strong**
			CO_2_ × Plant species	F_4_ = 0.71	0.59	None
Chlorogenic acid (n = 60)	buckwheat^1,2^, sunflower^1,2^, tomato^1^	lm(ln(chlorogenic acid) ~ CO_2_* plant_species + round + chamber)	CO_2_	F_1_ = 0.16	0.69	None
			Plant species	F_2_ = 29.23	** < 0.0001**	**Very strong**
			Round	F_1_ = 15.23	**0.0003**	**Strong**
			Chamber	F_1_ = 2.61	0.11	None
			CO_2_ × plant species	F_2_ = 4.58	**0.01**	**Strong**
Cinnamic acid (n = 48)	buckwheat^1,2^, squash^1,2^, sunflower^1^	lm(ln(cinnamic acid) ~ CO_2_* plant_species + round + chamber)	CO_2_	F_1_ = 0.93	0.34	None
			Plant species	F_2_ = 37.16	** < 0.0001**	**Very strong**
			Round	F_1_ = 5.71	**0.02**	**Strong**
			Chamber	F_1_ = 2.15	0.15	None
			CO_2_ × plant species	F_2_ = 0.52	0.6	None
Eugenol (n = 35)	buckwheat^2^, poppy^1^, sunflower^2^	lm(eugenol ~ CO_2_* Plant_species* species)**	CO_2_	F_1_ = 1.12	0.3	None
			Plant species	F_1_ = 2.24	0.15	None
			CO_2_ × plant species	F_1_ = 0.006	0.98	None
Gallic acid (n = 48)	buckwheat^1,2^, poppy^1^	lm(gallic acid ~ CO_2_* plant_species + round + chamber)	CO_2_	F_1_ = 0.52	0.47	None
			Plant species	F_1_ = 11.19	**0.002**	**Strong**
			Round	F_1_ = 7.02	**0.01**	**Strong**
			Chamber	F_1_ = 2.34	0.13	None
			CO_2_ × plant species	F_1_ = 0.13	0.72	None
Kaempferol (n = 117)	buckwheat^1,2^, poppy^1^, squash^1,2^, sunflower^1, 2^, tomato^1^	lm(ln(kaempferol) ~ CO_2_* plant_species + round)	CO_2_	F_1_ = 0.02	0.89	None
			Plant species	F_4_ = 102.96	** < 0.0001**	**Very strong**
			Round	F_1_ = 198.78	** < 0.0001**	**Very strong**
			Chamber	F_1_ = 0.22	0.64	None
			CO_2_ × plant species	F_4_ = 0.48	0.75	None
Nicotine (n = 10)	buckwheat^1^	*t*-test(nicotine ~ CO_2_)	CO_2_	*t*_6.75_ = -0.34	0.74	None
P-coumaric acid (n = 58)	buckwheat^1,2^, squash^2^, sunflower^2^	lm(ln(P-coumaric acid) ~ CO_2_* plant_species + round + chamber)	CO_2_	F_1_ = 0.24	0.62	None
			Plant species	F_2_ = 379.85	** < 0.0001**	**Very strong**
			Round	F_1_ = 153.69	** < 0.0001**	**Very strong**
			Chamber	F_1_ = 0.24	0.63	None
			CO_2_ × plant species	F_1_ = 1.06	0.35	None
Quercetin (n = 70)	buckwheat^1,2^, poppy^1^, squash^1^, sunflower^1^	lm(ln(quercetin) ~ CO_2_* plant_species + round + chamber)	CO_2_	F_1_ = 0.17	0.68	None
			Plant species	F_3_ = 256.46	** < 0.0001**	**Very strong**
			Round	F_1_ = 0.51	0.48	None
			Chamber	F_1_ = 10.06	**0.002**	**Strong**
			CO_2_ × plant species	F_3_ = 3.88	**0.01**	**Strong**

The superscript number(s) following a plant species’ common name indicates the experimental round(s) where data was included in analyses. For several metabolites, the metabolite quantity was natural-log transformed before linear model analysis to improve data normality as indicated by the model formula column. For linear models, the statistic value presented is the F-statistic, for the *t*-test, we report the t-statistic. Effect sizes are in Table S3 and species-level comparison were made using pairwise post-hoc tests summarized in Table S4. Bolded p-values indicate a significant finding (α = 0.05).

### *Experiment 2 – impacts of eCO*_*2*_* on pollen nutrition*

**Figure 3:**
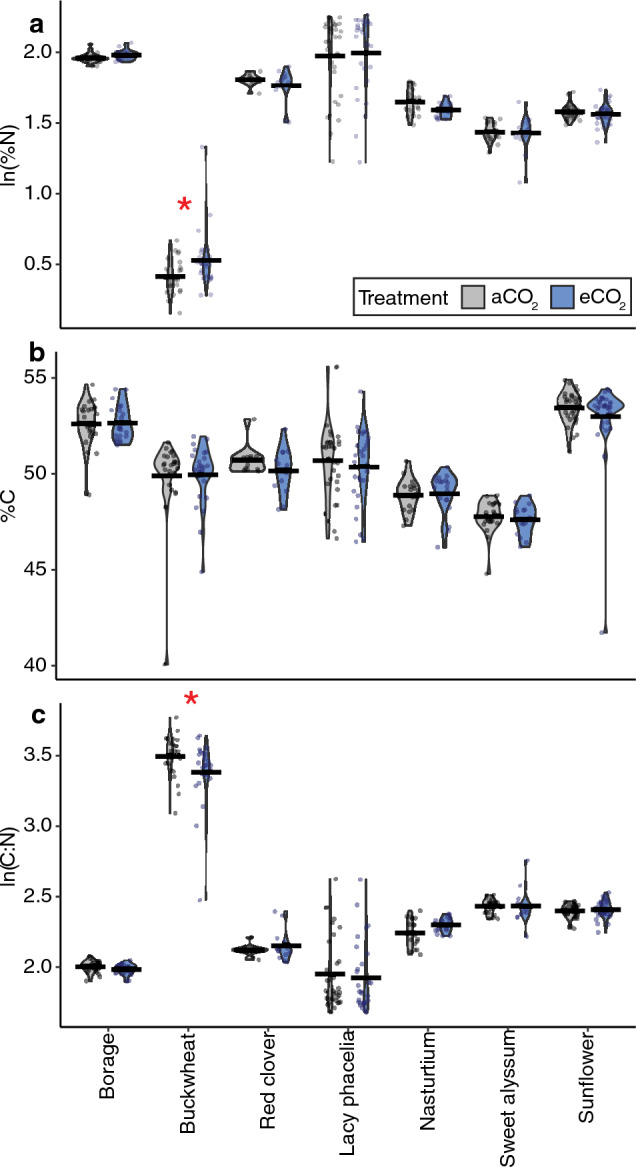
Impacts of elevated CO_2_ on pollen nutrition in Experiment 2. Pollen nutrition (both rounds pooled) from experiment 2 including %N (**a**), %C (**b**), and C:N ratio (**c**) for all studied plant species. Model details are in Table 2 with effect size and post-hoc comparisons in Tables S5 and S6, respectively. Significant differences between CO_2_ treatments are marked with a red asterisk (*).

*Nitrogen content.* We found very strong evidence that plant species (p < 0.0001) and strong evidence that experimental round (p = 0.01) affect %N, but no evidence that greenhouse chamber, CO_2_ treatment, or the interaction between plant species and CO_2_ affected pollen %N (Fig. 3a, statistical models summarized in Table 2; effect sizes in Table S5). Post-hoc tests revealed moderate evidence that eCO_2_ increased %N in buckwheat pollen (by 2.1%) (Table S6).

*Carbon content.* In the full model, we found very strong evidence that %C was affected by round and plant species (p < 0.0001 and < 0.0001, respectively), but no evidence that greenhouse chamber, CO_2_,﻿ nor the interaction between CO_2_ and species influenced %C (Fig. 3b, Table 2; Table S5). We found no evidence from post-hoc tests that eCO_2_ affected the %C in pollen for any individual species (Table S6).

*C:N ratios.* For C:N ratio in pollen, we found very strong evidence that plant species (p < 0.0001) and strong evidence that greenhouse chamber influenced C:N (p = 0.04) but no evidence that CO_2_ treatment, experimental round, or the interaction between CO_2_ and plant species affected C:N ratio (Fig. 3c, Table 2; Table S5). Post-hoc tests revealed strong evidence (p = 0.007) that the C:N ratio in buckwheat pollen decreased (by 9.7%) when grown in eCO_2_ (Table S6).

### Experiment 2–plant growth

**Figure 4:**
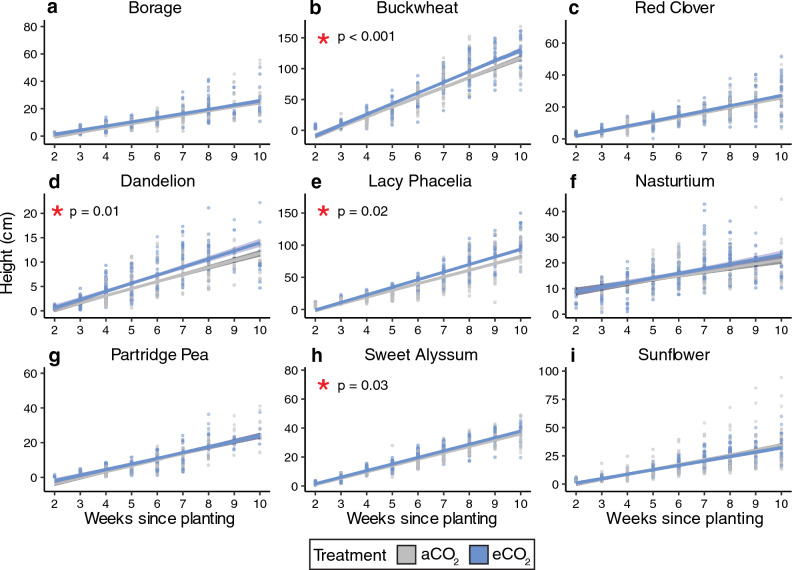
Height over time by plant species. Data from both rounds are pooled in this figure; lines are linear models. Plants with a significant effect of CO_2_ treatment on height are denoted with a red asterisk (*). Model outputs are in Table 4 and species-level comparisons are in Table S4.

*Plant height.* For plant height, we found moderate evidence that CO_2_ (p = 0.04) and very strong evidence that plant species, weeks since planting, and greenhouse chamber (all p-values < 0.0001) affected height with no evidence that experimental round or the interaction between CO_2_ and plant species influenced height (Table 4; effect sizes in Table S7). When comparing plant species with post-hoc tests, four species exhibited at least moderate evidence (all p ≤ 0.03) for an effect of CO_2_ on height, with five species, borage, red clover, nasturtium, sunflower and partridge pea, demonstrating no effect (Fig. 4, Table S8). At week 10, plants grown in eCO_2_, were on average, taller for four species: buckwheat (+ 19.6%), dandelion (+ 17.5%), lacy phacelia (+ 15.1%), and sweet alyssum (+ 17.7%).

**Table 4:** Plant growth and flowering characteristic analyses.

Variable	Model formula	Fixed effects	Statistic	p-value	Level of evidence
Height	lmer(ln(height) ~ CO_2_* plant_species + round + week + chamber + (1|plant_identity))	CO_2_	F_1,588.9_ = 4.10	**0.04**	**Moderate**
		Plant species	F_8,600.3_ = 481.67	** < 0.0001**	**Very strong**
		Round	F_1,644.3_ = 2.16	0.14	None
		Week	F_1,5733_ = 13,294.30	** < 0.0001**	**Very strong**
		Chamber	F_2,593.5_ = 15.60	** < 0.0001**	**Very strong**
		CO_2_ × plant species	F_8,599.5_ = 0.72	0.67	None
Leaf number	lmer(ln(leaves) ~ CO_2_* plant_species + round + week + chamber + (1|plant_identity))	CO_2_	F_1,731.9_ = 0.005	0.95	None
		Plant species	F_8,784.1_ = 285.43	** < 0.0001**	**Very strong**
		Round	F_1,795.4_ = 33.17	** < 0.0001**	**Very strong**
		Week	F_1,6056.9_ = 10,946.16	** < 0.0001**	**Very strong**
		Chamber	F_2,738.6_ = 2.5	0.08	Weak
		CO_2_ × plant species	F_8,745.7_ = 1.66	0.1	None
Flower number	glmer.nb(flower_no ~ CO_2_* plant_species + round + chamber + (1|week))	CO_2_	*X*^*2*^_1_ = 1.19	0.28	None
		Plant species	*X*^*2*^_7_ = 3728.95	** < 0.0001**	**Very strong**
		Round	*X*^*2*^_1_ = 15.29	** < 0.0001**	**Very strong**
		Chamber	*X*^*2*^_2_ = 10.13	**0.006**	**Strong**
		CO_2_ × plant species	*X*^*2*^_7_ = 35.61	** < 0.0001**	**Very strong**
Flowering initiation	lm(ln(flowering_start) ~ CO_2_* plant_species + round + chamber)	CO_2_	F_1_ = 3.29	0.07	Weak
		Plant species	F_7_ = 52.69	** < 0.0001**	**Very strong**
		Round	F_1_ = 0.16	0.69	None
		Chamber	F_2_ = 2	0.14	None
		CO_2_ × plant species	F_7_ = 1.81	0.08	Weak
Flower diameter	lm(ln(diameter) ~ CO_2_* plant_species + round + chamber)	CO_2_	F_1_ = 9.99	**0.002**	**Strong**
		Plant species	F_7_ = 4644.3	** < 0.0001**	**Very strong**
		Round	F_1_ = 0.48	0.49	None
		Chamber	F_2_ = 5.53	**0.004**	**Strong**
		CO_2_ × plant species	F_7_ = 1.09	0.37	None
Biomass	lm(ln(biomass) ~ CO_2_* plant_species + chamber)	CO_2_	F_1_ = 5.38	**0.02**	**Moderate**
		Plant species	F_8_ = 73.55	** < 0.0001**	**Very strong**
		Chamber	F_2_ = 3.66	**0.03**	**Moderate**
		CO_2_ × plant species	F_8_ = 2.73	**0.007**	**Strong**

For several growth measurements, the value was natural-log transformed before linear model analysis to improve data normality as indicated by the model formula column. For linear models, we report the F-statistic and for generalized linear mixed-models, we report the X^2^ statistic in the Statistics column. Species-level post-hoc comparisons are summarized in Table S8 with effect sizes in Tables S7, S9–S13. Bolded p-values indicate a significant finding (α = 0.05).

**Figure 5:**
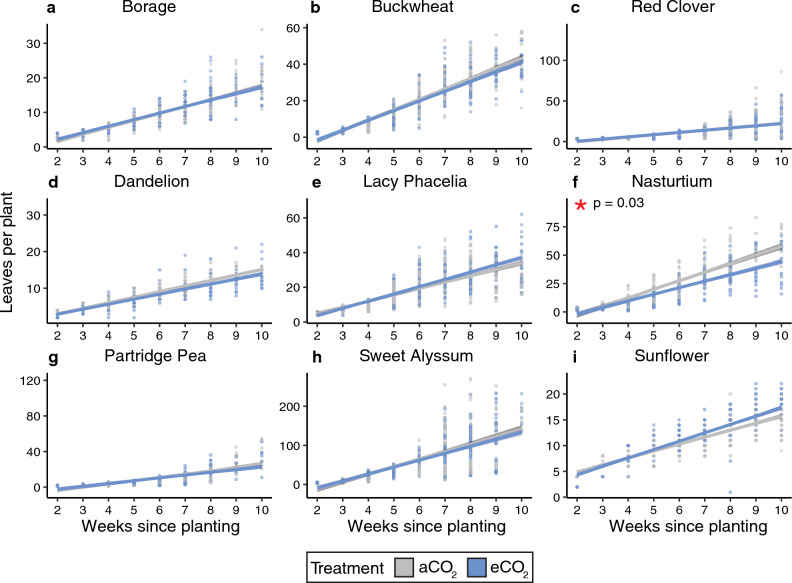
Number of leaves per plant by week for each species. Data from both experimental rounds are pooled in this figure; lines are linear models. Plants with a significant effect of CO_2_ treatment on leaf number are denoted with a red asterisk (*). Model outputs are in Table 4 and species-level comparisons are in Tables S8, S9.

*Leaf number.* We found strong evidence that plant species, experimental round, and week (all p < 0.0001), weak evidence that greenhouse chamber (p = 0.08), and no evidence that CO_2_ treatment, nor the interaction between plant species and CO_2_ treatment affected leaf number (Table 4; effect sizes in Table S9). When species were analyzed using post-hoc tests, we found moderate evidence that eCO_2_ reduced leaf number for nasturtium (p = 0.03; average change at week 10: −21%) (Fig. 5; Table S8).

**Figure 6:**
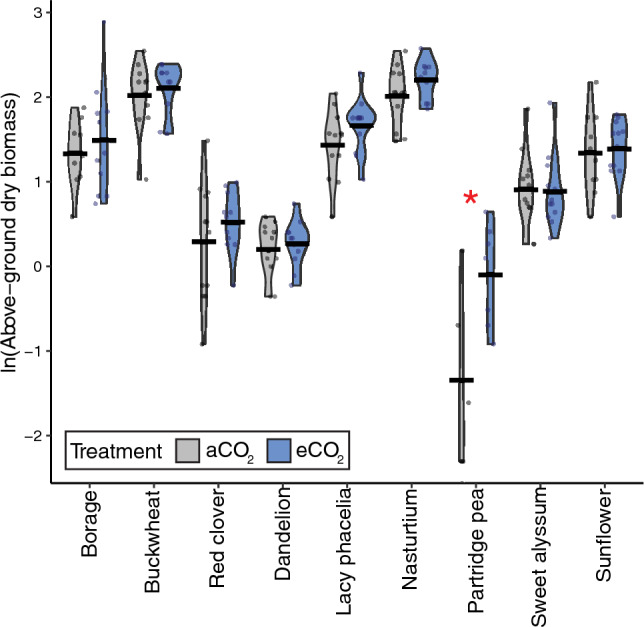
Above-ground dry biomass on day 60 after planting for plants in round 1 of experiment 2. Data from aCO_2_ conditions are presented in gray, while eCO_2_ is in blue. Significant effects of CO_2_ treatment on biomass are indicated with a red asterisk (*). Model outputs are in Table 4 with effect sizes in Table S10 and species-level comparisons are in Table S8.

*Biomass.* For above-ground biomass of plants in round 1, we found very strong evidence that plant species (p < 0.0001), strong evidence that the interaction between CO_2_ treatment and species affected biomass (p = 0.007), and moderate evidence that CO_2_ and greenhouse chamber influenced biomass (p = 0.02 and 0.03, respectively) (Table 4; effect sizes in Table S10). In post-hoc tests, partridge pea was the only species to show strong evidence (p < 0.001) for an effect of eCO_2_ on plant biomass (Fig. 6; Table S8), with plants grown under eCO_2_ having more (297%) biomass than those grown under aCO_2_.

**Figure 7:**
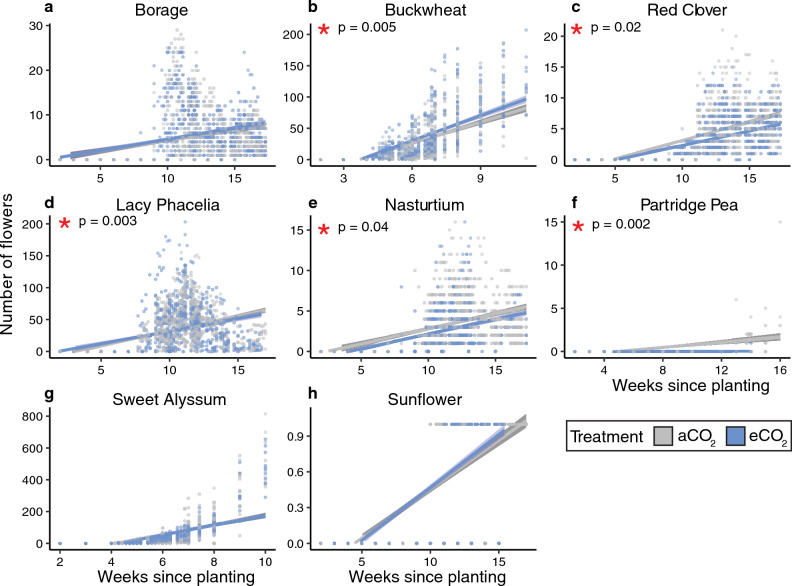
Number of flowers per plant by week for each species. Data from both experimental rounds are pooled in this figure; lines are linear models. Significant effects of CO_2_ treatment on biomass are indicated with a red asterisk (*). Model outputs are in Table 4, effect sizes in Table S11, and species-level comparisons are in Table S8.

*Flower number.* When we examined flower number across treatments and over time, we found very strong evidence that plant species, round, and the interaction between CO_2_ treatment and plant species (all p ≤ 0.0001; Table 4; effect sizes in Table S11) and strong evidence that greenhouse chamber (p = 0.006) affected flower number, with no evidence that CO_2_ did. When species were analyzed using post-hoc tests, we found very strong evidence that buckwheat, red clover, and nasturtium flower number (all p < 0.001; Table S8), strong evidence that partridge pea and lacy phacelia flower number (p = 0.006 and 0.01, respectively), and weak evidence that borage (p = 0.09) flower number was affected by CO_2_ treatment (Fig. 7; Table S8). Buckwheat and lacy phacelia grown in eCO_2_ had more flowers while red clover, nasturtium, and partridge pea plants had fewer, compared to those grown in aCO_2_ (Fig. 7).

**Figure 8:**
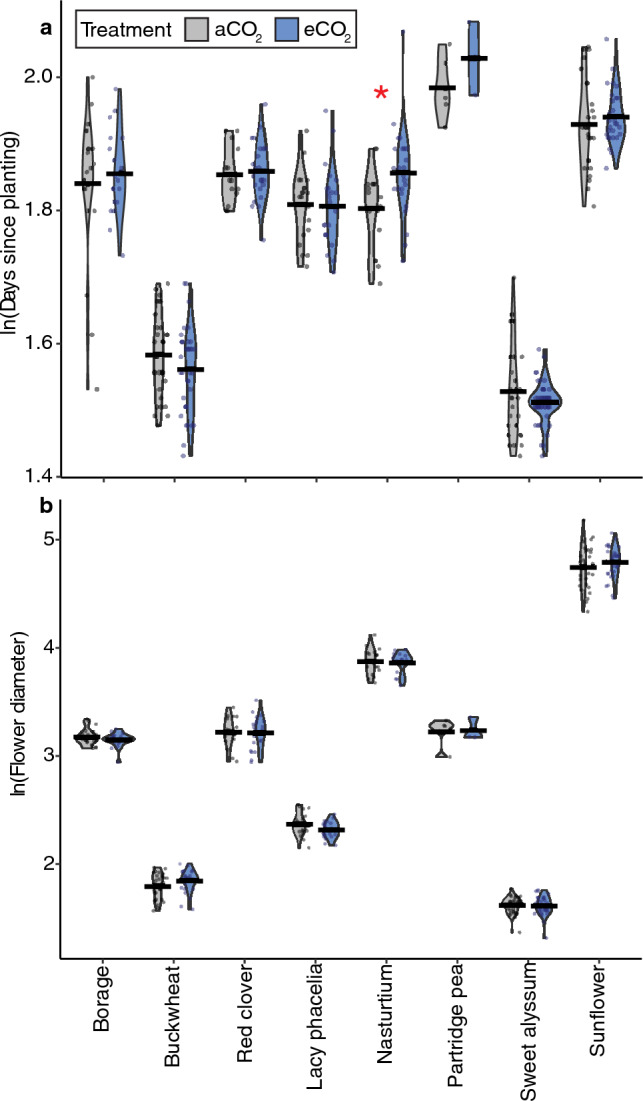
Days to first flower (**a**) and flower diameter (**b**) from plants in Experiment 2. Days to the first flower (**a**) and flower diameter (in mm) (**b**) for the plant species which flowered in experiment 2 (both rounds pooled). Significance (α = 0.05) is indicated in the figure above by a red asterisk (*). Model outputs are in Table 4, effect sizes in Tables S12, S13, and species-level comparisons are in Table S8.

*Flowering initiation.* We examined whether CO_2_ treatment influenced when plants began flowering (Fig. 8a) and found very strong evidence that plant species (p < 0.0001), weak evidence that CO_2_ (p = 0.07) and the interaction between CO_2_ and plant species (p = 0.08), but no evidence that round or greenhouse chamber affected flowering initiation (Table 4; effect sizes in Table S12). When species were analyzed separately with post-hoc tests, we found strong evidence (p = 0.001) that nasturtium flowered later in eCO_2_ (8.6 days later, 13.4%; Fig. 8a; Table S8).

*Flower diameter.* We found very strong evidence that flower diameter was affected by plant species (p < 0.0001), strong evidence that flower diameter was affected by CO_2_ treatment and greenhouse chamber (p = 0.002 and 0.004, respectively), and no evidence that the experimental round or the interaction between CO_2_ and plant species effected flower diameter (Fig. 8b; Table 4; effect sizes in Table S13). When plant species were analyzed separately using post-hoc tests, we found no evidence for an effect of CO_2_ on flower diameter for any species (Fig. 8b; Table S8).

### Functional group comparisons

We were unable to evaluate changes in pollen nutrition (%N) across either pair of functional groups (N-fixing ability, crop status) as only one species, buckwheat, exhibited a response to CO_2_ treatment (Table 5).

**Table 5:**
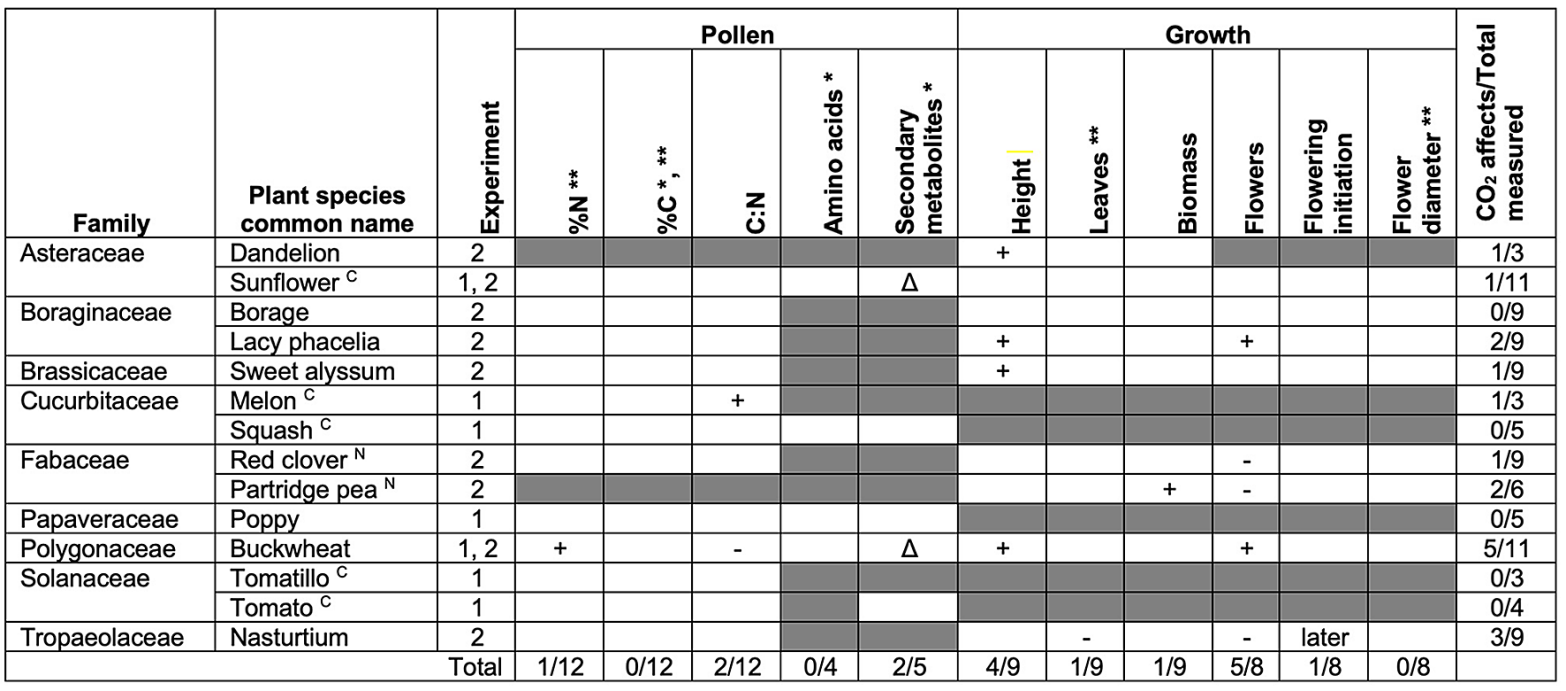
Summary of species-specific effects of eCO_2_ on plant growth and pollen chemistry.

To indicate plant functional traits, superscript letters following the plant species common name indicate plants that can fix N (^N^) and crop species (^C^). White boxes indicate a variable with no significant response to CO_2_ treatment. A grayed-out box indicates a response variable that was not measured for that plant species. Plus signs ( +) indicate where eCO_2_ led to a significant increase in a measured variable while minus signs (−) indicate the opposite. For pollen secondary metabolites, changes to one or more metabolites are denoted with a delta (Δ). Flowering initiation occurred significantly later for nasturtium plants grown under eCO_2_ and this is noted as “later”. Round was often a significant predictor of response variables; if a response variable was affected by round this is noted with a single asterisk for experiment 1 (*) and two asterisks for experiment 2 (**) following the variable name.

The two N-fixing species, red clover and partridge pea, did not exhibit consistent nor unique patterns in response to eCO_2_ (Table 5): partridge pea in eCO_2_ increased in biomass, but not height, while red clover in eCO_2_ demonstrated the opposite pattern: increased height but no change in biomass. Both species had fewer flowers in eCO_2_, but so did nasturtium, a non-N fixing plant.

When we compare the number of variables that responded to eCO_2_ between crop vs non-crop species, we found no evidence (Fisher’s exact test; p = 0.14) that crop plants (n = 5 species) responded to eCO_2_ (7.7% of the time) more or less often than non-crop plants (21.4%; n = 9 species) (Table 5).

## Discussion

We found species-specific effects on pollen composition and plant growth that were only partially consistent with our initial predictions, painting a complex picture of the effects of eCO_2_ on plant physiology, and, in turn, anticipated broader ecological effects. Across the variables we measured, and the plant species studied, some species and variables showed strong responses to eCO_2_ (i.e., buckwheat, plant height), while others demonstrated few or no responses (i.e., %C; sunflower) (Table 5). Pollen nutrition (%N), which we predicted would decrease for non-N fixing plants, did not change for the sole N-fixing plant (red clover) evaluated, nor for ten of eleven non-N fixing species (Figs. 1, 3; Table 5), requiring further study to elucidate potential trends across these functional groups. While we expected both pollen amino acids and secondary metabolites to change in response to eCO_2_, only two secondary metabolites (out of nine) were affected by CO_2_ treatment in up to two species (Table S4; Table 5). As we predicted, nearly half of the studied species studied here (44%) grew taller in eCO_2_ (Fig. 4). However, our prediction that flower number would not change across CO_2_ treatments was not true for most (63%) species (Fig. 7). Among species that did show significant effects of eCO_2_ on flower number, the direction of those responses diverged (3 species with fewer and 2 with more flowers). Plants in our study did not flower earlier or have changes in flower diameter when grown in eCO_2_, contrary to our prediction and previous research on other plant species^8,71,72^. The results here underscore the complexity of the effects of eCO_2_ on plant growth and physiology and are consistent with broad variability in CO_2_ responses found in the literature.

Pollen nutrition (%N, %C, and C:N) for only two plant species (buckwheat and melon) across both experiments were affected by CO_2_ treatment. Specifically, pollen %N (a direct proxy for pollen protein^33^) was only significantly affected by eCO_2_ for one plant species. Despite the increased carbon available in eCO_2_ growing conditions, we also found no evidence for carbon-loading (increased %C) in the pollen samples (Figs. 1b, 3b) nor many differences in C:N ratios (only buckwheat and melon; Fig. 1c**, **Fig. 3c) suggesting that eCO_2_ is not consistently altering the composition of compounds present in pollen (e.g., the ratio of C-rich:N-rich compounds was consistent across CO_2_ treatments) and is consistent with our findings from targeted metabolomic analyses (Fig. 2; Table 3). The general lack of C-loading in pollen we report here (Table 5) does not follow the well-documented trends of C-loading in other vegetative plant tissues^10–12^ nor the findings of Ziska et al.^17^, which found that under eCO_2_, goldenrod (*Solidago canadensis*) had higher C:N ratios, indicative of C-loading. Given the role of pollen in plant reproduction, pollen nutrition may exhibit stronger compensatory regulation than the chemistry of other plant tissues.

Changes to flowering patterns and floral reward nutrition are relevant for pollinator health and the critical pollination services they provide (e.g., flowering patterns^73^; pollen nutrition^74^). Our results suggest that the protein content of pollen may not show widespread changes under eCO_2_ (given the diverse and inconsistent responses across species; Fig. 3) but may alter the composition of amino acids in pollen (Fig. 2) and some secondary metabolites (Table 3). Although changes to amino acids were small (Fig. 2), amino acid composition structures bee foraging preferences^25,26^, therefore, even small changes to the relative abundance of these compounds in pollen could potentially modify pollinator floral preferences. When we investigated the effects of CO_2_ on secondary metabolites in pollen, which can guide associational memory in pollinators^24^ or chemically defend pollen^64,75,76^, we found that two chemical defense compounds, chlorogenic acid and quercetin, were affected by eCO_2_ in two plant species. Changes to pollen chemical defenses may reduce pollination success by making pollen grains more vulnerable to colonization by microbes or less competitive against conspecific pollen grains^76^. For pollinators, while the role of many secondary metabolites or amino acid composition in pollen are largely unknown, changes to these compounds could have important consequences for plant-pollinator interactions and require further study^77,78^.

In addition to the effects on pollen nutrition, other changes to flowering may have important consequences for pollinators. For example, changes to flowering time^8^ (i.e., later flowering by nasturtium, Fig. 8a) can result in phenological mismatches, where plants and pollinators are no longer active at the same time^32^. However, our findings on nasturtium flowering are inconsistent with another previous study on this species that found when exposed to 750 ppm eCO_2_ (vs. 380 CO_2_ ppm in aCO_2_ treatment) there was no difference in nasturtium flowering time (n = 43 plants^16^). The same study by Lake and Hughes^16^ found no difference in the number of flowers nasturtium produced, though our study found that this plant had fewer flowers under eCO_2_ (Fig. 7), though CO_2_ treatments and growing temperatures varied slightly between studies (day and night temperatures: 22 and 16 °C, respectively), which can influence CO_2_ responses^31,79^. In our study, red clover produced fewer flowers (a finding supported by Rusterholz and Erhardt^15^; n = 40 plants) as did partridge pea and nasturtium, but buckwheat and lacy phacelia plants produced more flowers under eCO_2_ (Fig. 7). Flower size changes may alter the potential pollinator community as changes to flower size may result in inaccessible floral resources for pollinators (i.e., flowers become too deep for pollinators to reach nectar, documented in bumble bees^80^). Here, we found no changes to flower size in any species (Fig. 8b), which contradicts findings for sunflower by Maia et al.^71^ where increased CO_2_ led to larger sunflower inflorescences (hybrid BRS 323; eCO_2_ of 800 ppm vs. 400 ppm in aCO_2_) and for pumpkin by Hoover et al.^72^ finding that flowers were smaller in eCO_2_ (Cucurbitaceae: *Cucurbita maxima* ‘Little Cutie’, eCO_2_ of 700 ppm vs. 360 ppm in aCO_2_). While we, as well as previous research, report changes to flowering patterns and floral rewards under eCO_2_^8^, these findings are not uniform across species (Table 5), making it hard to draw general predictions on how future CO_2_ levels are likely to impact pollinators via changes to their food resources. However, variable responses across species may still have important ecological impacts. For instance, when specialist bees are considered, they may be more vulnerable to these changes if their preferred floral resources are affected by eCO_2_ (e.g., goldenrod^17^, *Solidago* sp. specialists^81^).

We had initially predicted that in eCO_2_ growing conditions, other nutrients, like N, would become limited and thus result in reductions in N-rich compounds like proteins. Therefore, we included plant species in this study that can and cannot fix N (e.g., sunflowers cannot, while red clover can). We had high mortality rates with partridge pea, preventing us from including this species in pollen nutrition analyses and from comparing changes in %N across functional traits (N-fixers vs non-N-fixers). While red clover, which can fix N, did not show an effect of eCO_2_ on pollen %N, ten of eleven non-N-fixing plant species also were unaffected by eCO_2_ when pollen %N was considered (Table 5; Fig. 3a). However, these findings may be confounded by the nitrogen fertilization that we used. In the study by Ziska et al.^17^ that demonstrated the negative effects of eCO_2_ on pollen protein in goldenrod, plants were grown outside either as wild, unmanaged plants or, outdoors in FACE (Free-Atmospheric Carbon Experiment) plots without the addition of N fertilizer. As both experiments included in our study took place in a greenhouse setting, they required an addition of some N fertilizer to sustain plant growth in pots over time. While the feeding levels utilized in this study were low, it is possible that the addition of even limited N fertilizer was enough to overcome the negative impacts of eCO_2_ on pollen protein. The interactive effects of eCO_2_ and N fertilization on plant growth and pollen protein content are not well understood and warrant further investigation.

Crop species are often bred for characteristics desirable to humans like climate resilience^82^, palatability^83,84^, and quick growth^85^, and as such, may respond to environmental stimuli differently than their wild counterparts. When we considered crop and non-crop plants in this study, we found that non-crop plants were no more likely to respond to CO_2_ treatment than were crop species (Table 5), though for most crop species studied, we were only able to evaluate data on pollen chemistry and not growth. It is also possible that the crop plants, due to selective breeding, may already be growing at their limit and cannot grow faster even with increased CO_2_ availability. In contrast, wild plants, which have not been intentionally bred for fast growth, may still be able to leverage the excess CO_2_ to increase their growth. Furthermore, previous work has shown that crops grown in eCO_2_ experience a greater increase in fruits and seeds than wild species^7^, which was not investigated here. When crop species are bred into cultivars, they have a distinct set of traits^86–88^, which may not be present across cultivars, meaning that our findings on specific cultivars of crop species here may not be generalizable across cultivars. Future research investigating the role of eCO_2_ on crop species should incorporate cultivar to explore this relationship.

Given the well-documented positive relationship between increasing CO_2_ and plant biomass^30,31^, we expected to find an increase in above-ground biomass for all studied plant species. Here, only partridge pea demonstrated increased biomass when grown under eCO_2_ (Fig. 6) despite the increase in height for seven of nine species grown in eCO_2_ (Fig. 4; Table 5), and established positive relationship between plant height and biomass^89^. Height was repeatedly measured each week throughout the study allowing us to investigate trends over time, while biomass was measured once, on day 60, which may not have been enough time in eCO_2_ growing conditions for the species in this study to have significantly altered biomass. We report no effect of eCO_2_ on biomass for sunflowers, which contradicts the findings of two recent studies (hybrid BRS 323, eCO_2_: 800 ppm vs. aCO_2_: 400 ppm^71^; hybrid KBSH-1, eCO_2_: 550 or 700 ppm vs. aCO_2_: 380ppm^90^). However, our findings on dandelion biomass do align with findings from Thomas and Bazzaz^91^, which also found no change in dandelion biomass under eCO_2_ (700 ppm vs. aCO_2_: 350 ppm). While our findings on above-ground biomass partially align with existing literature, intraspecific responses to eCO_2_ are known^92^.

Responses to CO_2_ can be modulated by other environmental factors (e.g., soil nutrients^93^), which could account for some of the observed intraspecific differences between studies. For example, temperature is known to interact with CO_2_; in combination with warmer temperatures, eCO_2_ increases plant growth when compared to plants grown in either eCO_2_ or warmer temperatures (Solanaceae: *Capsicum* sp.^31^; Fabaceae: *Stylosanthes capitata*^79^). Here, experiment 2 took place during winter, which in northern North America, has relatively short amounts of daylight, and it is possible that variation in growth conditions (i.e., sunlight, temperatures) modulated plant responses to eCO_2_ in this study, even within a controlled greenhouse setting. Such modulation by environmental conditions would likely further complicate the challenge of predicting the effects of eCO_2_ on pollen nutrition and should be further investigated, especially given the future climate predictions of increased CO_2_, more extreme weather, and warmer temperatures^35^.

While plant responses to eCO_2_, both in the forms of growth and chemistry, are well-documented in the literature^2–8^, this study, in contrast, reports few responses to eCO_2_ that were often small and species-specific (Table 5). When considering all plant species together, this study found a significant effect of round for 70% (7 of 10) of the measured variables with data available across rounds (Table 5). The strong effect of round documented in this study indicates that even slight shifts in environmental conditions (e.g., planting dates six weeks apart even within a controlled, greenhouse environment) can influence plant growth. Notably, both studies described in this paper took place in greenhouses but outside of the natural growing season for North America (fall through early spring), and the corresponding reduced natural sunlight (despite grow lights in the greenhouse) and lower temperatures may have influenced plant responses to eCO_2_. In contrast, the study by Ziska et al.^17^ not only took place during the summer, but plants were grown wild, outdoors, and unconstrained by pots, which could also have impacted growth patterns and plant responses to eCO_2_. There may also be a CO_2_ saturation point where responses to eCO_2_ are diminished with increased CO_2_ exposure, which could also explain differential findings across studies and should be considered in future research. Another potential contributor to differential responses in plant growth could be differences in sample sizes, or relatively small sample sizes (e.g., n of ~ 40 plants per species^15,16^ vs n of ~ 48–96 plants per species in this study; Table 1) possibly leading to divergent results. Lastly, the duration of CO_2_ exposure also appears to affect the directionality and size of plant responses. For example, Silva et al.^18^ found that in black maple, very short (6 h) exposure to eCO_2_ (500, 1000, 3000 ppm) either did not affect, or increased pollen protein, but exposure to the same CO_2_ treatments for 24 h, led to a decrease in pollen protein. The findings from our study, when contextualized within the existing literature, underscore the sensitivity of plants to CO_2_ levels and the complexity of studying the effects of eCO_2_ on plant growth and chemistry.

A potential limitation of our study design, which measured multiple individual plants within greenhouse chambers, is the possibility of pseudoreplication^94^. In ecology, potential non-independence of data (e.g., arising from blocked treatments or sampling) is a widespread challenge^94–96^ and can lead to spurious findings of false positive results (i.e., finding a significant relationship when there isn’t one, or Type I error). However, there is mixed evidence for the importance of pseudoreplication^95–99^, with some authors arguing that sampling many individuals within a population may result in non-independence among samples, but not necessarily pseudoreplication^100^, or that measuring multiple individuals within a given experimental setting can help to hone estimates of variance^101^. To account for potential sources of non-independence in our data, we performed multiple experimental rounds and included greenhouse chamber as a fixed effect in all models to assess chamber-level variance. Given that we report few significant responses of eCO_2_ by any plant species studied here (17 responses out of 96 measured), we consider it unlikely that we are over-reporting the impacts of CO_2_ on plant growth or pollen nutrition.

Overall, we documented the effects of eCO_2_ growing conditions on the growth habits, and pollen nutrition of fourteen flowering plant species spanning phylogenetic and functional diversity, the largest species-level evaluation of plant characteristics to date. Responses to eCO_2_ are small where present and vary across species. Pollen protein does not appear to uniformly decrease across the species studied here, nor are other flowering traits consistently affected. While our findings suggest that elevated atmospheric CO_2_ may not have widespread, consistent negative effects on pollinator nutrition, divergent responses across species may still have important effects on plant-pollinator communities, as well as particular (e.g., specialist) pollinator taxa. Similarly, patterns in plant growth under eCO_2_ were only consistent for plant height. The findings of this study highlight the need for further studies exploring how plant growth and floral nutrition are likely to change in a future with increasing levels of CO_2_ and the consequences for plant-pollinator interactions.

### Supplementary Information

Supplementary Information.

## Data Availability

All data produced in this study can be found on Dryad (https://doi.org/10.5061/dryad.70rxwdc5n). Code used for analysis and figure-making can be found on GitHub (https://github.com/Crall-Lab/eCO2_SciReports.git).
