# Supplementary material for: Elevated atmospheric CO2 has small, species-specific effects on pollen chemistry and plant growth across flowering plant species
Source: Sci Rep. 2024 Jun 14;14:13760. doi: 10.1038/s41598-024-63967-z (PMC11178917; doi:10.1038/s41598-024-63967-z)
Supplement: Supplementary file 1 — Supplementary Information. [file 41598_2024_63967_MOESM1_ESM.docx]

ELECTRONIC SUPPLEMENTARY MATERIAL

Olivia M. Bernauer, Anupreksha Jain, Benjamin de Bivort, N. Michele Holbrook, Samuel S. Myers, Lewis H. Ziska, James D. Crall. **Elevated atmospheric CO_2_ has small, species-specific effects on pollen chemistry and plant growth across flowering plant species**. *Scientific Reports* in revision, resubmitted 14 May 2024.

TABLE OF CONTENTS

**Figure S1:** CO_2_ levels by treatment across rounds. 2

**Table S1:** Effect sizes for pollen nutrition samples from experiment 1. 3

**Table S2:** Post-hoc pairwise comparisons by plant species across CO_2_ treatments for

experiment 1 pollen nutrition data. 4

**Table S3:** Effect sizes for pollen secondary metabolites from experiment 1. 5

**Table S4:** Secondary metabolite post-hoc pairwise comparisons by plant species across

CO_2_ treatments in experiment 1. *7*

**Table S5:** Effect sizes for pollen nutrition samples from experiment 2. 8

**Table S6:** Pollen chemistry post-hoc pairwise comparisons by plant species across

CO_2_ treatments for experiment 2. 9

**Table S7:** Effect sizes for plant height in experiment 2. 10

**Table S8:** Post-hoc pairwise comparisons by plant species across CO_2_ treatments on plant

growth and flowering characteristics in experiment 2. 11

**Table S9:** Effect sizes for leaf number in experiment 2. 12

**Table S10:** Effect sizes for above-ground biomass in experiment 2. 13

**Table S11:** Effect sizes for above-ground biomass in experiment 2. 14

**Table S12:** Effect sizes for flowering initiation in experiment 2. 15

**Table S13:** Effect sizes for flower diameter in experiment 2. 16

##### Figure S1: CO_2_ levels by treatment across rounds.

*CO_2_ values during experiments 1 (a) and 2 (b) separated by experimental round.*

**
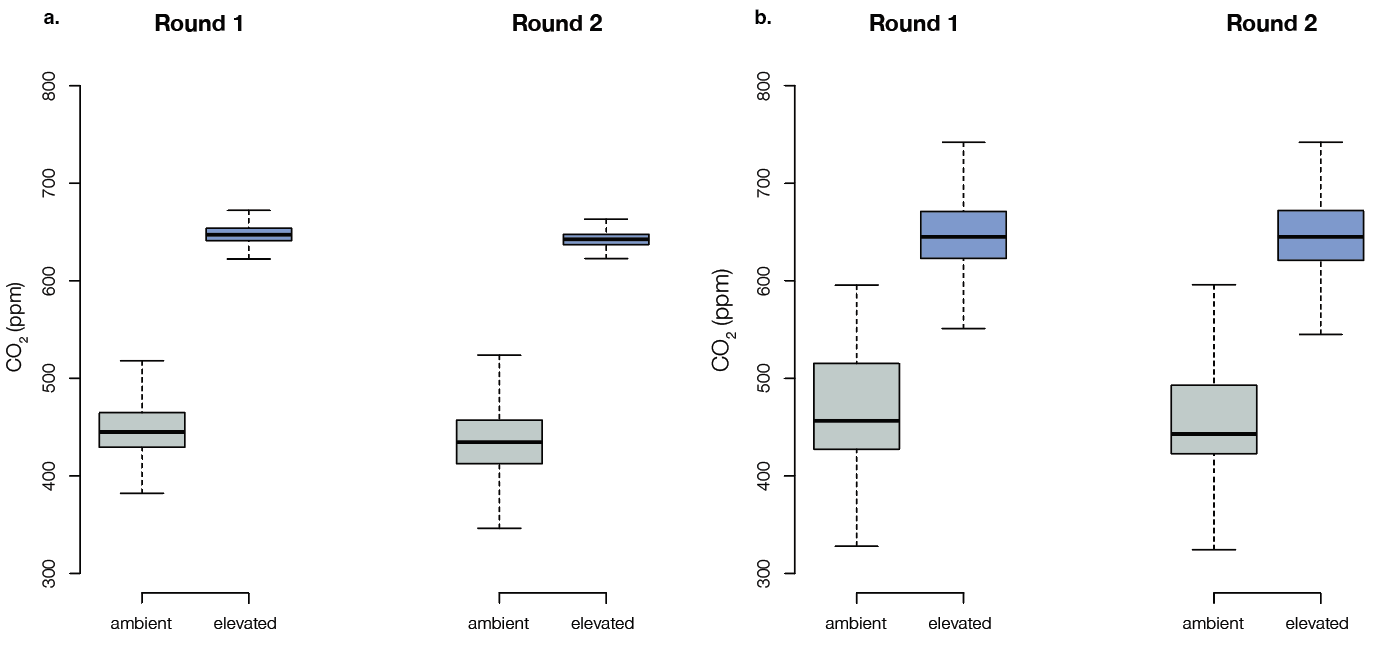
**

#####

#####

**Table S1: Effect sizes for pollen nutrition samples from experiment 1.**

*Model outputs are detailed in* ***Table 2*** *with pairwise species-level comparisons in* ***Table S2****.*

| **Variable** |  | **Estimate** | **Std. Error** | **p-value** |
| --- | --- | --- | --- | --- |
| %N | (Intercept) | 4.10 | 0.23 | < 0.0001 |
|  | CO_2_ (elevated) | -0.51 | 0.35 | 0.15 |
|  | Plant (poppy) | 2.07 | 0.26 | < 0.0001 |
|  | Plant (squash) | 1.41 | 0.28 | < 0.0001 |
|  | Plant (sunflower) | 0.29 | 0.27 | 0.28 |
|  | Plant (tomatillo) | 4.05 | 0.26 | < 0.0001 |
|  | Plant (tomato) | 4.29 | 0.29 | < 0.0001 |
|  | Round 2 | -0.21 | 0.12 | 0.09 |
|  | Chamber 2 | 0.03 | 0.12 | 0.82 |
|  | CO_2_ (elevated) x Plant (poppy) | 0.54 | 0.37 | 0.15 |
|  | CO_2_ (elevated) x Plant (squash) | 0.64 | 0.41 | 0.12 |
|  | CO_2_ (elevated) x Plant (sunflower) | 0.27 | 0.40 | 0.5 |
|  | CO_2_ (elevated) x Plant (tomatillo) | 0.71 | 0.37 | 0.06 |
|  | CO_2_ (elevated) x Plant (tomato) | 0.51 | 0.41 | 0.21 |
| %C | (Intercept) | 41.89 | 0.41 | < 0.0001 |
|  | CO_2_ (elevated) | -0.54 | 0.62 | 0.39 |
|  | Plant (poppy) | 6.97 | 0.46 | < 0.0001 |
|  | Plant (squash) | 7.49 | 0.5 | < 0.0001 |
|  | Plant (sunflower) | 9.29 | 0.48 | < 0.0001 |
|  | Plant (tomatillo) | 6.2 | 0.45 | < 0.0001 |
|  | Plant (tomato) | 6.70 | 0.51 | < 0.0001 |
|  | Round 2 | -0.99 | 0.22 | < 0.0001 |
|  | Chamber 2 | -0.06 | 0.22 | 0.8 |
|  | CO_2_ (elevated) x Plant (poppy) | 0.38 | 0.66 | 0.56 |
|  | CO_2_ (elevated) x Plant (squash) | 0.56 | 0.72 | 0.44 |
|  | CO_2_ (elevated) x Plant (sunflower) | 0.68 | 0.7 | 0.33 |
|  | CO_2_ (elevated) x Plant (tomatillo) | 0.01 | 0.66 | 0.99 |
|  | CO_2_ (elevated) x Plant (tomato) | 0.63 | 0.72 | 0.38 |
| C:N | (Intercept) | 10.23 | 0.32 | < 0.0001 |
|  | CO_2_ (elevated) | 1.28 | 0.49 | 0.01 |
|  | Plant (poppy) | -2.28 | 0.36 | < 0.0001 |
|  | Plant (squash) | -1.29 | 0.39 | 0.001 |
|  | Plant (sunflower) | 1.45 | 0.38 | 0.0002 |
|  | Plant (tomatillo) | -4.32 | 0.35 | < 0.0001 |
|  | Plant (tomato) | -4.44 | 0.4 | < 0.0001 |
|  | Round 2 | 0.17 | 0.17 | 0.33 |
|  | Chamber 2 | 0.05 | 0.17 | 0.75 |
|  | CO_2_ (elevated) x Plant (poppy) | -1.41 | 0.52 | 0.007 |
|  | CO_2_ (elevated) x Plant (squash) | -1.48 | 0.56 | 0.01 |
|  | CO_2_ (elevated) x Plant (sunflower) | -0.74 | 0.55 | 0.18 |
|  | CO_2_ (elevated) x Plant (tomatillo) | -1.56 | 0.51 | 0.003 |
|  | CO_2_ (elevated) x Plant (tomato) | -1.35 | 0.56 | 0.02 |

##### Table S2: Post-hoc pairwise comparisons by plant species across CO_2_ treatments for experiment 1 pollen nutrition data.

*Model outputs are in* ***Table 2****; effect sizes in* ***Table S1****. A bolded p-value indicates significance (𝛼 < 0.05).*

| **Variable** | **Plant species** | **t ratio** | **p-value** | **Level of evidence** |
| --- | --- | --- | --- | --- |
| %N | Melon | *t*_96_ = 1.45 | 0.15 | none |
|  | Poppy | *t*_96_ = -0.14 | 0.89 | none |
|  | Squash | *t*_96_ = -0.74 | 0.46 | none |
|  | Sunflower | *t*_96_ = 1.1 | 0.28 | none |
|  | Tomatillo | *t*_96_ = -0.95 | 0.35 | none |
|  | Tomato | *t*_96_ = 0.02 | 0.99 | none |
| %C | Melon | *t*_96_ = 0.87 | 0.39 | none |
|  | Poppy | *t*_96_ = 0.51 | 0.61 | none |
|  | Squash | *t*_96_ = -0.06 | 0.96 | none |
|  | Sunflower | *t*_96_ = -0.36 | 0.72 | none |
|  | Tomatillo | *t*_96_ = 1.44 | 0.15 | none |
|  | Tomato | *t*_96_ = -0.19 | 0.85 | none |
| C:N | Melon | *t*_96_ = -2.63 | **0.01** | **strong** |
|  | Poppy | *t*_96_ = 0.52 | 0.61 | none |
|  | Squash | *t*_96_ = 0.79 | 0.43 | none |
|  | Sunflower | *t*_96_ = -1.77 | 0.08 | none |
|  | Tomatillo | *t*_96_ = 0.94 | 0.35 | none |
|  | Tomato | *t*_96_ = 0.18 | 0.86 | none |

#####

**Table S3:** **Effect sizes for pollen secondary metabolites from experiment 1.**

*Model outputs are detailed in* ***Table 3*** *with pairwise species-level comparisons in* ***Table S4****.*

| **Secondary metabolite** |  | **Estimate** | **Std. Error** | **p-value** |
| --- | --- | --- | --- | --- |
| Caffeine | (Intercept) | 12.63 | 0.45 | < 0.0001 |
|  | CO_2_ (elevated) | 0.50 | 0.51 | 0.33 |
|  | Plant (poppy) | 0.41 | 0.42 | 0.34 |
|  | Plant (squash) | 0.51 | 0.36 | 0.15 |
|  | Plant (sunflower) | 0.14 | 0.33 | 0.67 |
|  | Plant (tomato) | 0.23 | 0.48 | 0.63 |
|  | Round 2 | -1.75 | 0.24 | < 0.0001 |
|  | Chamber | 0.57 | 0.24 | 0.02 |
|  | CO_2_ (elevated) x Plant (poppy) | -0.61 | 0.66 | 0.35 |
|  | CO_2_ (elevated) x Plant (squash) | -0.80 | 0.67 | 0.23 |
|  | CO_2_ (elevated) x Plant (sunflower) | -0.35 | 0.60 | 0.57 |
|  | CO_2_ (elevated) x Plant (tomato) | -1.01 | 0.74 | 0.17 |
| Chlorogenic acid | (Intercept) | 17.61 | 0.73 | < 0.0001 |
|  | CO_2_ (elevated) | -1.6 | 0.46 | 0.001 |
|  | Plant (sunflower) | -4.31 | 0.5 | < 0.0001 |
|  | Plant (tomato) | -3.47 | 0.66 | < 0.0001 |
|  | Round 2 | -1.36 | 0.42 | 0.002 |
|  | Chamber | -0.20 | 0.42 | 0.63 |
|  | CO_2_ (elevated) x Plant (sunflower) | 2.49 | 0.83 | 0.004 |
|  | CO_2_ (elevated) x Plant (tomato) | 1.94 | 0.97 | 0.05 |
| Cinnamic acid | (Intercept) | 10.65 | 0.45 | < 0.0001 |
|  | CO_2_ (elevated) | -0.29 | 0.3 | 0.34 |
|  | Plant (squash) | -1.80 | 0.33 | < 0.0001 |
|  | Plant (sunflower) | -2.18 | 0.44 | < 0.0001 |
|  | Round2 | -0.67 | 0.26 | 0.02 |
|  | Chamber | 0.34 | 0.26 | 0.21 |
|  | CO_2_ (elevated) x Plant (squash) | 0.46 | 0.48 | 0.35 |
|  | CO_2_ (elevated) x Plant (sunflower) | 0.31 | 0.63 | 0.62 |
| Eugenol | (Intercept) | 1292414 | 317190 | 0.0003 |
|  | CO_2_ (elevated) | -306300 | 448574 | 0.5 |
|  | Plant (poppy) | 476150 | 437216 | 0.28 |
|  | CO_2_ (elevated) x Plant (poppy) | 15230 | 648940 | 0.98 |
| Gallic acid | (Intercept) | -2139866 | 6649020 | 0.75 |
|  | CO_2_ (elevated) | -2210885 | 3106175 | 0.48 |
|  | Plant (poppy) | 11844636 | 4739699 | 0.02 |
|  | Round 2 | 8118071 | 3106175 | 0.01 |
|  | Chamber | 2492375 | 3106175 | 0.43 |
|  | CO_2_ (elevated) x Plant (poppy) | 2195128 | 6165814 | 0.72 |
|  |  |  |  |  |
| **Secondary metabolite** |  | **Estimate** | **Std. Error** | **p-value** |
| Kaempferol | (Intercept) | 18.6852 | 0.41 | < 0.0001 |
|  | CO_2_ (elevated) | -0.29 | 0.33 | 0.38 |
|  | Plant (poppy) | -0.32 | 0.4 | 0.43 |
|  | Plant (squash) | -1.67 | 0.34 | < 0.0001 |
|  | Plant (sunflower) | -3.91 | 0.31 | < 0.0001 |
|  | Plant (tomato) | -1.82 | 0.46 | 0.0001 |
|  | Round 2 | -2.79 | 0.2 | < 0.0001 |
|  | Chamber | 0.15 | 0.2 | 0.45 |
|  | CO_2_ (elevated) x Plant (poppy) | -0.14 | 0.57 | 0.80 |
|  | CO_2_ (elevated) x Plant (squash) | 0.30 | 0.48 | 0.53 |
|  | CO_2_ (elevated) x Plant (sunflower) | 0.42 | 0.46 | 0.36 |
|  | CO_2_ (elevated) x Plant (tomato) | -0.17 | 0.65 | 0.79 |
| P-coumaric acid | (Intercept) | 12.94 | 0.51 | < 0.0001 |
|  | CO_2_ (elevated) | 0.25 | 0.27 | 0.35 |
|  | Plant (squash) | -5.94 | 0.33 | < 0.0001 |
|  | Plant (sunflower) | -5.91 | 0.38 | < 0.0001 |
|  | Round2 | 3.30 | 0.27 | < 0.0001 |
|  | Chamber | 0.03 | 0.27 | 0.91 |
|  | CO_2_ (elevated) x Plant (squash) | -0.03 | 0.45 | 0.94 |
|  | CO_2_ (elevated) x Plant (sunflower) | -0.71 | 0.52 | 0.18 |
| Quercetin | (Intercept) | 18.94 | 0.66 | < 0.0001 |
|  | CO_2_ (elevated) | -1.07 | 0.34 | 0.003 |
|  | Plant (poppy) | -7.67 | 0.54 | < 0.0001 |
|  | Plant (squash) | -5.40 | 0.59 | < 0.0001 |
|  | Plant (sunflower) | -6.32 | 0.48 | < 0.0001 |
|  | Round2 | 0.24 | 0.34 | 0.48 |
|  | Chamber | -0.05 | 0.34 | 0.89 |
|  | CO_2_ (elevated) x Plant (poppy) | 2.06 | 0.73 | 0.006 |
|  | CO_2_ (elevated) x Plant (squash) | 1.23 | 0.88 | 0.17 |
|  | CO_2_ (elevated) x Plant (sunflower) | 2.22 | 0.7 | 0.002 |

##### Table S4: Secondary metabolite post-hoc pairwise comparisons by plant species across CO_2_ treatments in experiment 1.

*Model outputs are in* ***Table 3*** *with effect sizes in* ***Table S3****. Both rounds are pooled for analyses. A bolded p-value indicates significance (𝛼 < 0.05).*

| **Variable** | **Plant species** | **t-ratio** | **p-value** | **Level of evidence** |
| --- | --- | --- | --- | --- |
| Caffeine  n = 108 | Buckwheat | *t*_96_ = -0.98 | 0.33 | none |
|  | Poppy | *t*_96_ = 0.23 | 0.82 | none |
|  | Squash | *t*_96_ = 0.76 | 0.45 | none |
|  | Sunflower | *t*_96_ = -0.48 | 0.63 | none |
|  | Tomato | *t*_96_ = 0.88 | 0.38 | none |
| Chlorogenic acid  n = 60 | Buckwheat | *t*_52_ = 3.44 | **0.001** | **strong** |
|  | Sunflower | *t*_52_ = -1.41 | 0.17 | none |
|  | Tomato | *t*_52_ = -0.43 | 0.66 | none |
| Cinnamic acid  n = 48 | Buckwheat | *t*_40_ = 0.97 | 0.34 | none |
|  | Squash | *t*_40_ - -0.42 | 0.68 | none |
|  | Sunflower | *t*_40_ = -0.03 | 0.97 | none |
| Eugenol  n = 35 | Buckwheat | *t*_31_ = 0.68 | 0.5 | none |
|  | Poppy | *t*_31_ = 0.62 | 0.54 | none |
| Gallic acid  n = 48 | Buckwheat | *t*_41_ = 0.71 | 0.48 | none |
|  | Poppy | *t*_41_ = 0.004 | 0.99 | none |
| Kaempferol  n = 117 | Buckwheat | *t*_105_ = 0.88 | 0.38 | none |
|  | Poppy | *t*_105_ = 0.99 | 0.33 | none |
|  | Squash | *t*_105_ = -0.04 | 0.97 | none |
|  | Sunflower | *t*_105_ = -0.42 | 0.67 | none |
|  | Tomato | *t*_105_ = 0.86 | 0.39 | none |
| P-coumaric acid  n = 58 | Buckwheat | *t*_50_ = -0.95 | 0.35 | none |
|  | Squash | *t*_50_ = -0.51 | 0.61 | none |
|  | Sunflower | *t*_50_ = 0.92 | 0.36 | none |
| Quercetin  n = 70 | Buckwheat | *t*_60_ = 3.12 | **0.002** | **strong** |
|  | Poppy | *t*_60_ = -1.71 | 0.09 | none |
|  | Squash | *t*_60_ = -0.21 | 0.83 | none |
|  | Sunflower | *t*_60_ = -2.13 | **0.04** | **strong** |

#####

**Table S5: Effect sizes for pollen nutrition samples from experiment 2**

*Model outputs are detailed in* ***Table 2*** *with pairwise species-level comparisons in* ***Table S6****.*

| **Variable** |  | **Estimate** | **Std. Error** | **p-value** |
| --- | --- | --- | --- | --- |
| %N | (Intercept) | 1.91 | 0.04 | < 0.0001 |
|  | CO_2_ (elevated) | 0.01 | 0.05 | 0.75 |
|  | Plant (buckwheat) | -1.55 | 0.04 | < 0.0001 |
|  | Plant (clover) | -0.15 | 0.05 | 0.004 |
|  | Plant (lacy phacelia) | 0.006 | 0.04 | 0.89 |
|  | Plant (nasturtium) | -0.31 | 0.05 | < 0.0001 |
|  | Plant (sweet alyssum) | -0.53 | 0.04 | < 0.0001 |
|  | Plant (sunflower) | -0.39 | 0.04 | < 0.0001 |
|  | Round | 0.05 | 0.02 | 0.008 |
|  | Chamber 63 | -0.03 | 0.02 | 0.24 |
|  | Chamber 62 | -0.04 | 0.02 | 0.07 |
|  | CO_2_ (elevated) x Plant (buckwheat) | 0.08 | 0.06 | 0.16 |
|  | CO_2_ (elevated) x Plant (clover) | -0.06 | 0.07 | 0.37 |
|  | CO_2_ (elevated) x Plant (lacy phacelia) | 0.005 | 0.06 | 0.93 |
|  | CO_2_ (elevated) x Plant (nasturtium) | -0.1 | 0.06 | 0.14 |
|  | CO_2_ (elevated) x Plant (sweet alyssum) | -0.03 | 0.07 | 0.66 |
|  | CO_2_ (elevated) x Plant (sunflower) | -0.04 | 0.06 | 0.51 |
| %C | (Intercept) | 51.26 | 0.40 | < 0.0001 |
|  | CO_2_ (elevated) | -0.06 | 0.45 | 0.89 |
|  | Plant (buckwheat) | -2.84 | 0.41 | < 0.0001 |
|  | Plant (clover) | -1.70 | 0.51 | 0.0009 |
|  | Plant (lacy phacelia) | -2.02 | 0.39 | < 0.0001 |
|  | Plant (nasturtium) | -3.63 | 0.44 | < 0.0001 |
|  | Plant (sweet alyssum) | -4.79 | 0.42 | < 0.0001 |
|  | Plant (sunflower) | 0.76 | 0.39 | 0.05 |
|  | Round | 0.86 | 0.17 | < 0.0001 |
|  | Chamber 63 | 0.33 | 0.23 | 0.15 |
|  | Chamber 62 | 0.06 | 0.22 | 0.78 |
|  | CO_2_ (elevated) x Plant (buckwheat) | -0.15 | 0.58 | 0.8 |
|  | CO_2_ (elevated) x Plant (clover) | -0.91 | 0.71 | 0.2 |
|  | CO_2_ (elevated) x Plant (lacy phacelia) | -0.37 | 0.56 | 0.51 |
|  | CO_2_ (elevated) x Plant (nasturtium) | -0.31 | 0.63 | 0.62 |
|  | CO_2_ (elevated) x Plant (sweet alyssum) | -0.19 | 0.64 | 0.76 |
|  | CO_2_ (elevated) x Plant (sunflower) | -0.53 | 0.55 | 0.34 |
| C:N | (Intercept) | 2.02 | 0.04 | < 0.0001 |
|  | CO_2_ (elevated) | -0.02 | 0.04 | 0.72 |
|  | Plant (buckwheat) | 1.5 | 0.04 | < 0.0001 |
|  | Plant (clover) | 0.12 | 0.05 | 0.02 |
|  | Plant (lacy phacelia) | -0.05 | 0.04 | 0.23 |
|  | Plant (nasturtium) | 0.24 | 0.04 | < 0.0001 |
|  | Plant (sweet alyssum) | 0.43 | 0.04 | < 0.0001 |
|  | Plant (sunflower) | 0.40 | 0.04 | < 0.0001 |
|  | Round | -0.03 | 0.02 | 0.08 |
|  | Chamber 63 | 0.03 | 0.02 | 0.13 |
|  | Chamber 62 | 0.04 | 0.02 | 0.05 |
|  | CO_2_ (elevated) x Plant (buckwheat) | -0.09 | 0.06 | 0.13 |
|  | CO_2_ (elevated) x Plant (clover) | 0.05 | 0.07 | 0.51 |
|  | CO_2_ (elevated) x Plant (lacy phacelia) | -0.01 | 0.05 | 0.82 |
|  | CO_2_ (elevated) x Plant (nasturtium) | 0.09 | 0.06 | 0.15 |
|  | CO_2_ (elevated) x Plant (sweet alyssum) | 0.02 | 0.06 | 0.7 |
|  | CO_2_ (elevated) x Plant (sunflower) | 0.03 | 0.05 | 0.63 |

##### Table S6: Pollen chemistry post-hoc pairwise comparisons by plant species across CO_2_ treatments for experiment 2.

*Model outputs are in* ***Table 2*** *with effect sizes in* ***Table S5****. A bolded p-value indicates significance (𝛼 < 0.05).*

| **Variable** | **Plant species** | **t-ratio** | **p-value** | **Level of evidence** |
| --- | --- | --- | --- | --- |
| %N | Borage | *t*_311_ = -0.51 | 0.61 | none |
|  | Buckwheat | *t*_311_ = -2.56 | **0.01** | **moderate** |
|  | Red Clover | *t*_311_ = 0.04 | 0.47 | none |
|  | Lacy Phacelia | *t*_311_ = -0.71 | 0.48 | none |
|  | Nasturtium | *t*_311_ = 1.54 | 0.13 | none |
|  | Sweet alyssum | *t*_311_ = 0.13 | 0.9 | none |
|  | Sunflower | *t*_311_ = 0.41 | 0.68 | none |
| %C | Borage | *t*_311_ = -0.18 | 0.86 | none |
|  | Buckwheat | *t*_311_ = 0.19 | 0.85 | none |
|  | Red Clover | *t*_311_ = 1.47 | 0.14 | none |
|  | Lacy Phacelia | *t*_311_ = 0.8 | 0.42 | none |
|  | Nasturtium | *t*_311_ = 0.5 | 0.62 | none |
|  | Sweet alyssum | *t*_311_ = 0.25 | 0.8 | none |
|  | Sunflower | *t*_311_ = 1.25 | 0.21 | none |
| C:N | Borage | *t*_311_ = 0.5 | 0.62 | none |
|  | Buckwheat | *t*_311_ = 2.7 | **0.007** | **strong** |
|  | Red Clover | *t*_311_ = -0.46 | 0.65 | none |
|  | Lacy Phacelia | *t*_311_ = 0.9 | 0.37 | none |
|  | Nasturtium | *t*_311_ = -1.5 | 0.13 | none |
|  | Sweet alyssum | *t*_311_ = -0.08 | 0.93 | none |
|  | Sunflower | *t*_311_ = -0.17 | 0.87 | none |

##### Table S7: Effect sizes for plant height in experiment 2.

*Model outputs are detailed in* ***Table 4*** *with pairwise species-level comparisons in* ***Table S8****.*

|  | **Estimate** | **Std. Error** | **p-value** |
| --- | --- | --- | --- |
| (Intercept) | 0.963 | 0.061 | < 0.0001 |
| CO_2_ (elevated) | 0.052 | 0.071 | 0.4599 |
| Plant (buckwheat) | 1.218 | 0.065 | < 0.0001 |
| Plant (clover) | 0.127 | 0.063 | 0.0452 |
| Plant (dandelion) | -0.949 | 0.066 | < 0.0001 |
| Plant (lacy phacelia) | 1.219 | 0.063 | < 0.0001 |
| Plant (nasturtium) | 0.289 | 0.071 | < 0.0001 |
| Plant (partridge pea) | -0.468 | 0.075 | < 0.0001 |
| Plant (sweet alyssum) | 0.356 | 0.065 | < 0.0001 |
| Plant (sunflower) | 0.294 | 0.063 | < 0.0001 |
| Round | -0.032 | 0.022 | 0.1424 |
| Week | 0.225 | 0.002 | < 0.0001 |
| Chamber (63) | -0.032 | 0.030 | 0.2912 |
| Chamber (62) | -0.155 | 0.030 | < 0.0001 |
| CO_2_ (elevated) x Plant (buckwheat) | 0.104 | 0.092 | 0.2594 |
| CO_2_ (elevated) x Plant (clover) | -0.021 | 0.089 | 0.8166 |
| CO_2_ (elevated) x Plant (dandelion) | 0.052 | 0.093 | 0.578 |
| CO_2_ (elevated) x Plant (lacy phacelia) | 0.030 | 0.090 | 0.7377 |
| CO_2_ (elevated) x Plant (nasturtium) | -0.045 | 0.099 | 0.6495 |
| CO2(elevated) x Plant (partridge pea) | 0.005 | 0.105 | 0.959 |
| CO_2_ (elevated) x Plant (sweet alyssum) | 0.023 | 0.091 | 0.797 |
| CO_2_ (elevated) x Plant (sunflower) | -0.075 | 0.089 | 0.4028 |

##### Table S8: Post-hoc pairwise comparisons by plant species across CO_2_ treatments on plant growth and flowering characteristics in experiment 2.

*Model outputs are in* ***Table 4*** *with effect sizes in* ***Table S7****. A bolded p-value indicates significance (𝛼 < 0.05).*

| **Variable** | **Plant species** | **t-ratio/z-ratio** | **p-value** | **Level of evidence** |
| --- | --- | --- | --- | --- |
| Height | Borage | *t*_755_ = -1.65 | 0.1 | none |
|  | Buckwheat | *t*_808_ = -3.40 | **0.0007** | **very strong** |
|  | Red Clover | *t*_726_ = -1.56 | 0.12 | none |
|  | Dandelion | *t*_884_ = -2.54 | **0.01** | **strong** |
|  | Lacy Phacelia | *t*_751_ = -2.39 | **0.02** | **moderate** |
|  | Nasturtium | *t*_791_ = -0.92 | 0.36 | none |
|  | Partridge Pea | *t*_902_ = -1.45 | 0.15 | none |
|  | Sweet alyssum | *t*_758_ = -2.23 | **0.03** | **moderate** |
|  | Sunflower | *t*_731_ = -0.65 | 0.52 | none |
| Leaf number | Borage | *t*_677_ = -0.25 | 0.80 | none |
|  | Buckwheat | *t*_775_ = -0.24 | 0.81 | none |
|  | Red Clover | *t*_649_ = -1 | 0.32 | none |
|  | Dandelion | *t*_810_ = 1.88 | 0.06 | weak |
|  | Lacy Phacelia | *t*_675_ = 0.07 | 0.94 | none |
|  | Nasturtium | *t*_719_ = 2.14 | **0.03** | **moderate** |
|  | Partridge Pea | *t*_835_ = 0.61 | 0.54 | none |
|  | Sweet alyssum | *t*_772_ = -1.78 | 0.08 | weak |
|  | Sunflower | *t*_657_ = -0.7 | 0.48 | none |
| Flower number | Borage | z = -1.51 | 0.13 | none |
|  | Buckwheat | z = -2.87 | **0.005** | **strong** |
|  | Red Clover | z = 2.38 | **0.02** | **moderate** |
|  | Lacy Phacelia | z = -3.01 | **0.003** | **strong** |
|  | Nasturtium | z = 2.04 | **0.04** | **moderate** |
|  | Partridge Pea | z = 3.1 | **0.002** | **strong** |
|  | Sweet alyssum | z = -0.16 | 0.88 | none |
|  | Sunflower | z = 0.43 | 0.67 | none |
| Flowering initiation | Borage | *t*_356_ = -0.3 | 0.77 | none |
|  | Buckwheat | *t*_356_ = 0.86 | 0.39 | none |
|  | Red Clover | *t*_356_ = -0.37 | 0.71 | none |
|  | Lacy Phacelia | *t*_356_ = 0.43 | 0.67 | none |
|  | Nasturtium | *t*_356_ = -3.22 | **0.001** | **strong** |
|  | Partridge Pea | *t*_356_ = -1.52 | 0.13 | none |
|  | Sweet alyssum | *t*_356_ = 0.57 | 0.57 | none |
|  | Sunflower | *t*_356_ = -0.66 | 0.51 | none |
| Flower diameter | Borage | *t*_330_ = 0.73 | 0.47 | none |
|  | Buckwheat | *t*_330_ = -1.60 | 0.11 | none |
|  | Red Clover | *t*_330_ = -0.12 | 0.91 | none |
|  | Lacy Phacelia | *t*_330_ = 1.40 | 0.16 | none |
|  | Nasturtium | *t*_330_ = 0.17 | 0.87 | none |
|  | Partridge Pea | *t*_330_ = -0.19 | 0.85 | none |
|  | Sweet alyssum | *t*_330_ = 0.21 | 0.83 | none |
|  | Sunflower | *t*_330_ = -1.62 | 0.11 | none |
| Biomass | Borage | *t*_173_ = -0.98 | 0.33 | none |
|  | Buckwheat | *t*_173_ = -0.51 | 0.61 | none |
|  | Red Clover | *t*_173_ = -1.26 | 0.21 | none |
|  | Dandelion | *t*_173_ = -0.39 | 0.7 | none |
|  | Lacy Phacelia | *t*_173_ = -1.3 | 0.2 | none |
|  | Nasturtium | *t*_173_ = -0.98 | 0.33 | none |
|  | Partridge Pea | *t*_173_ = -5.12 | **< 0.001** | **very strong** |
|  | Sweet alyssum | *t*_173_ = 0.1 | 0.92 | none |
|  | Sunflower | *t*_173_ = -0.33 | 0.74 | none |

##### Table S9: Effect sizes for leaf number in experiment 2.

*Model outputs are detailed in* ***Table 4*** *with pairwise species-level comparisons in* ***Table S8****.*

|  | **Estimate** | **Std. Error** | **p-value** |
| --- | --- | --- | --- |
| (Intercept) | 0.88 | 0.06 | < 0.0001 |
| CO_2_ (elevated) | 0.03 | 0.07 | 0.68 |
| Plant (buckwheat) | 0.51 | 0.06 | < 0.0001 |
| Plant (clover) | 0.09 | 0.06 | 0.12 |
| Plant (dandelion) | 0.06 | 0.06 | 0.36 |
| Plant (lacy phacelia) | 0.69 | 0.06 | < 0.0001 |
| Plant (nasturtium) | 0.77 | 0.07 | < 0.0001 |
| Plant (partridge pea) | -0.16 | 0.07 | 0.02 |
| Plant (sweet alyssum) | 1.41 | 0.06 | < 0.0001 |
| Plant (sunflower) | 0.18 | 0.06 | 0.002 |
| Round | -0.12 | 0.02 | < 0.0001 |
| Week | 0.22 | 0.002 | < 0.0001 |
| Chamber (63) | -0.06 | 0.03 | 0.05 |
| Chamber (62) | -0.03 | 0.03 | 0.26 |
| CO_2_ (elevated) x Plant (buckwheat) | -0.001 | 0.09 | 0.99 |
| CO_2_ (elevated) x Plant (clover) | 0.04 | 0.08 | 0.64 |
| CO_2_ (elevated) x Plant (dandelion) | -0.13 | 0.09 | 0.14 |
| CO_2_ (elevated) x Plant (lacy phacelia) | -0.02 | 0.08 | 0.82 |
| CO_2_ (elevated) x Plant (nasturtium) | -0.16 | 0.09 | 0.08 |
| CO_2_ (elevated) x Plant (partridge pea) | -0.06 | 0.1 | 0.53 |
| CO_2_ (elevated) x Plant (sweet alyssum) | 0.09 | 0.09 | 0.31 |
| CO_2_ (elevated) x Plant (sunflower) | 0.02 | 0.08 | 0.79 |

##### Table S10: Effect sizes for above-ground biomass in experiment 2.

*Model outputs are detailed in* ***Table 4*** *with pairwise species-level comparisons in* ***Table S8****.*

|  | **Estimate** | **Std. Error** | **p-value** |
| --- | --- | --- | --- |
| (Intercept) | 1.16 | 0.15 | < 0.0001 |
| CO_2_ (elevated) | 0.31 | 0.21 | 0.14 |
| Plant (buckwheat) | 0.70 | 0.19 | 0.0004 |
| Plant (clover) | -1.01 | 0.19 | < 0.0001 |
| Plant (dandelion) | -1.10 | 0.19 | < 0.0001 |
| Plant (lacy phacelia) | 0.13 | 0.19 | 0.49 |
| Plant (nasturtium) | 0.71 | 0.19 | 0.0003 |
| Plant (partridge pea) | -2.73 | 0.24 | < 0.0001 |
| Plant (sweet alyssum) | -0.4 | 0.19 | 0.04 |
| Plant (sunflower) | 0.02 | 0.19 | 0.90 |
| Chamber (61) | 0.28 | 0.09 | 0.002 |
| Chamber (63) | 0.05 | 0.09 | 0.59 |
| CO_2_ (elevated) x Plant (buckwheat) | -0.09 | 0.27 | 0.74 |
| CO_2_ (elevated) x Plant (clover) | 0.04 | 0.27 | 0.87 |
| CO_2_ (elevated) x Plant (dandelion) | -0.12 | 0.27 | 0.66 |
| CO_2_ (elevated) x Plant (lacy phacelia) | 0.05 | 0.26 | 0.86 |
| CO_2_ (elevated) x Plant (nasturtium) | -0.003 | 0.27 | 0.99 |
| CO_2_ (elevated) x Plant (partridge pea) | 1.15 | 0.32 | 0.0005 |
| CO_2_ (elevated) x Plant (sweet alyssum) | -0.21 | 0.26 | 0.43 |
| CO_2_ (elevated) x Plant (sunflower) | -0.13 | 0.27 | 0.63 |

**Table S11: Effect sizes for flower number in experiment 2.**

*Model outputs are detailed in* ***Table 4*** *with pairwise species-level comparisons in* ***Table S8****.*

|  | **Estimate** | **Std. Error** | **p-value** |
| --- | --- | --- | --- |
| (Intercept) | -2.44 | 1.37 | 0.07 |
| CO_2_ (elevated) | 0.07 | 0.17 | 0.67 |
| Plant (buckwheat) | 5.78 | 0.17 | < 0.0001 |
| Plant (clover) | -0.39 | 0.16 | 0.02 |
| Plant (lacy phacelia) | 1.92 | 0.14 | < 0.0001 |
| Plant (nasturtium) | -0.36 | 0.17 | 0.03 |
| Plant (partridge pea) | -1.65 | 0.27 | < 0.0001 |
| Plant (sweet alyssum) | 6.6 | 0.18 | < 0.0001 |
| Plant (sunflower) | -2.64 | 0.21 | < 0.0001 |
| Round | 0.27 | 0.07 | < 0.0001 |
| Chamber (63) | 0.09 | 0.08 | 0.28 |
| Chamber (62) | -0.25 | 0.08 | 0.003 |
| CO_2_ (elevated) x Plant (buckwheat) | 0.11 | 0.21 | 0.59 |
| CO_2_ (elevated) x Plant (clover) | -0.61 | 0.22 | 0.01 |
| CO_2_ (elevated) x Plant (lacy phacelia) | 0.13 | 0.20 | 0.51 |
| CO_2_ (elevated) x Plant (nasturtium) | -0.63 | 0.25 | 0.01 |
| CO_2_ (elevated) x Plant (partridge pea) | -3.50 | 1.08 | 0.001 |
| CO_2_ (elevated) x Plant (sweet alyssum) | -0.22 | 0.21 | 0.30 |
| CO_2_ (elevated) x Plant (sunflower) | -0.35 | 0.31 | 0.25 |

**Table S12: Effect sizes for flowering initiation in experiment 2.**

*Model outputs are detailed in* ***Table 4*** *with pairwise species-level comparisons in* ***Table S8****.*

|  | **Estimate** | **Std. Error** | **p-value** |
| --- | --- | --- | --- |
| (Intercept) | 3.22 | 0.03 | < 0.0001 |
| CO_2_ (elevated) | -0.05 | 0.04 | 0.16 |
| Plant (buckwheat) | -1.38 | 0.03 | < 0.0001 |
| Plant (clover) | 0.03 | 0.04 | 0.36 |
| Plant (lacy phacelia) | -0.81 | 0.04 | < 0.0001 |
| Plant (nasturtium) | 0.69 | 0.04 | < 0.0001 |
| Plant (partridge pea) | 0.04 | 0.06 | 0.52 |
| Plant (sweet alyssum) | -1.56 | 0.03 | < 0.0001 |
| Plant (sunflower) | 1.566923 | 0.03 | < 0.0001 |
| Round | -0.009 | 0.01 | 0.47 |
| Chamber (63) | -0.005 | 0.02 | 0.79 |
| Chamber (62) | -0.06 | 0.02 | 0.001 |
| CO_2_ (elevated) x Plant (buckwheat) | 0.08 | 0.05 | 0.11 |
| CO_2_ (elevated) x Plant (clover) | 0.03 | 0.05 | 0.56 |
| CO_2_ (elevated) x Plant (dandelion) | -0.02 | 0.05 | 0.63 |
| CO_2_ (elevated) x Plant (lacy phacelia) | 0.02 | 0.05 | 0.72 |
| CO_2_ (elevated) x Plant (nasturtium) | 0.04 | 0.09 | 0.65 |
| CO_2_ (elevated) x Plant (partridge pea) | 0.02 | 0.05 | 0.66 |
| CO_2_ (elevated) x Plant (sweet alyssum) | 0.07 | 0.05 | 0.11 |

**Table S13: Effect sizes for flower diameter in experiment 2.**

*Model outputs are detailed in* ***Table 4*** *with pairwise species-level comparisons in* ***Table S8****.*

|  | **Estimate** | **Std. Error** | **p-value** |
| --- | --- | --- | --- |
| (Intercept) | 4.25 | 0.04 | < 0.0001 |
| CO_2_ (elevated) | 0.05 | 0.04 | 0.26 |
| Plant (buckwheat) | -0.59 | 0.04 | < 0.0001 |
| Plant (clover) | 0.03 | 0.05 | 0.53 |
| Plant (lacy phacelia) | -0.07 | 0.04 | 0.1 |
| Plant (nasturtium) | -0.09 | 0.04 | 0.05 |
| Plant (partridge pea) | 0.33 | 0.06 | < 0.0001 |
| Plant (sweet alyssum) | -0.72 | 0.04 | < 0.0001 |
| Plant (sunflower) | 0.20 | 0.04 | < 0.0001 |
| Round | -0.006 | 0.02 | 0.68 |
| Chamber (63) | -0.04 | 0.02 | 0.07 |
| Chamber (62) | 0.003 | 0.02 | 0.88 |
| CO_2_ (elevated) x Plant (buckwheat) | -0.08 | 0.05 | 0.13 |
| CO_2_ (elevated) x Plant (clover) | -0.02 | 0.06 | 0.76 |
| CO_2_ (elevated) x Plant (lacy phacelia) | -0.04 | 0.06 | 0.55 |
| CO_2_ (elevated) x Plant (nasturtium) | 0.09 | 0.06 | 0.14 |
| CO_2_ (elevated) x Plant (partridge pea) | 0.05 | 0.11 | 0.62 |
| CO_2_ (elevated) x Plant (sweet alyssum) | -0.07 | 0.05 | 0.22 |
| CO_2_ (elevated) x Plant (sunflower) | -0.006 | 0.06 | 0.92 |
